# Novel Schemes of No-Slip Boundary Conditions for the Discrete Unified Gas Kinetic Scheme Based on the Moment Constraints

**DOI:** 10.3390/e25050780

**Published:** 2023-05-10

**Authors:** Wenqiang Guo, Guoxiang Hou

**Affiliations:** 1 Hypervelocity Aerodynamics Institute, China Aerodynamics Research and Development Center, Mianyang 621000, China; 15927101347@163.com; 2 Laboratory of Aerodynamics in Multiple Flow Regimes, China Aerodynamics Research and Development Center, Mianyang 621000, China; 3 School of Naval Architecture and Ocean Engineering, Huazhong University of Science and Technology, Wuhan 430074, China

**Keywords:** DUGKS, boundary condition, moment-based scheme, dipole–wall collision, Rayleigh–Taylor instability

## Abstract

The boundary conditions are crucial for numerical methods. This study aims to contribute to this growing area of research by exploring boundary conditions for the discrete unified gas kinetic scheme (DUGKS). The importance and originality of this study are that it assesses and validates the novel schemes of the bounce back (BB), non-equilibrium bounce back (NEBB), and Moment-based boundary conditions for the DUGKS, which translate boundary conditions into constraints on the transformed distribution functions at a half time step based on the moment constraints. A theoretical assessment shows that both present NEBB and Moment-based schemes for the DUGKS can implement a no-slip condition at the wall boundary without slip error. The present schemes are validated by numerical simulations of Couette flow, Poiseuille flow, Lid-driven cavity flow, dipole–wall collision, and Rayleigh–Taylor instability. The present schemes of second-order accuracy are more accurate than the original schemes. Both present NEBB and Moment-based schemes are more accurate than the present BB scheme in most cases and have higher computational efficiency than the present BB scheme in the simulation of Couette flow at high Re. The present Moment-based scheme is more accurate than the present BB, NEBB schemes, and reference schemes in the simulation of Poiseuille flow and dipole–wall collision, compared to the analytical solution and reference data. Good agreement with reference data in the numerical simulation of Rayleigh–Taylor instability shows that they are also of use to the multiphase flow. The present Moment-based scheme is more competitive in boundary conditions for the DUGKS.

## 1. Introduction

Discrete Unified Gas Kinetic Scheme (DUGKS) for all Knudsen Number Flows was proposed [1], which combined the advantages of the Lattice Boltzmann method (LBM) and the Unified Gas Kinetic Scheme (UGKS). The DUGKS and LBM can share the same equilibrium distribution function, and both start from the Boltzmann equation. Although the computational time cost of DUGKS is slightly higher than that of LBM in a continuous flow, the numerical stability of DUGKS is better than that of LBM, and it can capture flow characteristics that LBM is not competent to [2,3]. Both DUGKS and UGKS have asymptotic preserving properties, where the time step is not limited by the collision time. For the reconstruction of cell-interface flux, the UGKS adopts the analytical time-evolved integral solution and the DUGKS adopts a simpler numerical characteristic solution, in which the calculation time cost is approximately 70% of that in the UGKS [4]. In short, the DUGKS has a simple framework and the powerful ability to capture the flow characteristics in a wide flow regime, which can make the DUGKS a competitive tool in comparison with LBM and UGKS. Recently, the DUGKS has been successfully applied to a variety of flow problems in different flow regimes, such as turbulent flows [5,6,7,8], micro flows [9,10,11,12,13], compressible flows [4,14,15,16,17], multiphase flows [18,19,20], fluid-particle flows [21,22], flows with complex geometries [23,24], gas mixture systems [25,26], pore-scale porous media flows [27], and plasma physics [28,29]. In addition to flow problems, the DUGKS was also extended to multiscale transport problems such as phonon heat transfer [30,31,32] and the radiation of photons [33,34].

The boundary conditions are crucial for numerical methods [35,36,37]. If boundary conditions are not properly introduced in the numerical methods, severe problems may arise, such as the divergence of numerical solutions. In short, boundary conditions should be treated with great care. Because the LBM and DUGKS share a common kinetic origin, researchers tried to introduce the boundary conditions from the LBM into the DUGKS. For example, two kinds of no-slip boundary conditions are widely used: The bounce-back (BB) scheme [1] and the non-equilibrium bounce-back (NEBB) scheme [38]. They have received considerable critical attention. For the LBM and DUGKS, there are distinctive modeling differences in the particle evolution process. The LBM separates the particle streaming and collision process. However, particle transport and collision are fully coupled in DUGKS. This dynamic difference can result in the deviation of the boundary condition in the LBM and DUGKS. For example, the boundary condition is processed at time *t* + 0.5Δ*t* in the DUGKS but at time *t* in the LBM. Yang et al. [39] have analyzed and assessed the BB scheme and the NEBB scheme for the DUGKS. Although the BB scheme is a simple and common method to apply the no-slip boundary condition, it introduces an additional error (a purely artificial numerical slip error) into the DUGKS. The NEBB scheme eliminates the error of the numerical slip, but the closure to find the unknowns at the wall is somewhat arbitrary [40]. There are only a few studies on boundary conditions for the DUGKS, which have not been studied thoroughly. So, it can bring renewed interest and value to introduce and test other boundary conditions for the DUGKS.

Recently, the moment-based boundary condition has received increased interest and attention [40,41,42,43,44,45], which is based on the moments of the LBM [40] and has not yet been introduced to the DUGKS. Numerical simulations show that the moment-based boundary condition converges with second-order accuracy using dipole–wall collisions [40], natural convection in the square cavity [41], and lid-driven cavity flow [43,44,45]. A numerical simulation of the Poiseuille flow shows that the moment-based boundary condition can eliminate the spurious oscillations seen in solutions using other boundary conditions, considering the nonzero deviatoric stress [42]. The moment-based boundary condition can be parallelized easily for efficient computing, and it is conceptually simpler and more straightforward than other methods that involve a mixture of the bounce-back rule, hydrodynamic moments, momentum corrections, and other modifications to the distribution functions [44].

To the best of the authors’ knowledge, the moment-based boundary condition for the DUGKS has not been analyzed. So, the present work introduces the moment-based boundary condition to the DUGKS, and its numerical performance is studied in comparison with the BB scheme and the NEBB scheme. Generally, for boundary conditions in the DUGKS, the boundary nodes are located at the cell interface, and the unknown original distribution functions are treated at time *t_n_*_+1/2_ = *t_n_* + 0.5Δ*t* [39]. In the DUGKS, the original distribution functions are obtained after the transformed distribution functions are calculated. So, in this study, novel schemes of the BB, NEBB, and Moment-based boundary conditions are proposed for the DUGKS, which treat the unknown transformed distribution functions at time *t_n_*_+1/2_ = *t_n_* + 0.5Δ*t*. That is, the implementation of the boundary condition shifts from the original f(***x****_w_*, ***ξ****_α_*, *t* + *h*) to the transformed $\bar{f}$(***x****_w_*, ***ξ****_α_*, *t* + *h*), which may improve the numerical performance.

Assessment and validation of the novel schemes are necessary and important to their application. To test boundary conditions for the DUGKS, numerical simulations have only focused on the Couette flow and the Poiseuille flow in Ref. [39]. Considering the generality, more complex flows should be adopted to test boundary conditions, such as the multiphase flow. The multiphase flows are of both academic and industrial interest [46,47,48,49,50,51,52], such as the unsteady Rayleigh–Taylor instability. The benchmark problem of Rayleigh–Taylor instability has been used to validate the newly developed numerical approaches [18,19,50,51]. The bounce-back boundary conditions are applied to the bottom and top wall boundaries in the DUGKS simulations of Rayleigh–Taylor instability [18]. However, to the best of the authors’ knowledge, the effect of varied wall boundary conditions on the numerical simulation of Rayleigh–Taylor instability has been not investigated. Therefore, in this work, the unsteady Rayleigh–Taylor instability is used to test and validate the proposed schemes of boundary conditions, in addition to the Couette flow, the Poiseuille flow, the dipole–wall collision, and the lid-driven cavity flow.

The rest of the paper is organized as follows. In Section 2, the DUGKS with a force term, the original and novel schemes of BB, NEBB, and Moment-based boundary conditions for the DUGKS are present. In Section 3, the boundary conditions are tested and assessed using Couette flow, Poiseuille flow, Lid-driven cavity flow, vortex dipole–wall collision, and Rayleigh–Taylor instability. In Section 4, the conclusions are drawn.

## 2. Numerical Method

It is noted that the present work is for isothermal continuum flow.

### 2.1. DUGKS with a Force Term

In the LBM, a modeled gas, which is composed of identical particles whose velocities are restricted to a finite set of vectors, is considered. Similar to the LBM, the DUGKS follows the lattice Boltzmann equation. It is inevitably necessary to introduce a force term to the DUGKS in some cases, such as the external force driven by Poiseuille flow. The governing equation of the DUGKS with a force term [3]:

(1) $$\frac{\partial f}{\partial t}+\xi_{\alpha }\cdot \nabla f=\Omega +S$$

Equation (1) describes the spatial and temporal evolution of the distribution *f* (***ξ****_α_*, ***x***, *t*) of particles with velocity ***ξ****_α_* at position ***x*** and time *t*. The fundamental variable in gas kinetic theory is the particle distribution function, which simultaneously represents the density of mass in both physical space and velocity space.

The commonly used Bhatnagar–Gross–Krook (BGK) model is given as

(2) $$\Omega =(f^{eq}-f)/\tau$$

where *τ* denotes the relaxation time.

The force term is approximated as [3]

(3) $$S=-a\cdot \nabla_{\xi }f\approx a\cdot (\xi -u)f^{eq}/RT$$

where ***a*** denotes the acceleration due to the external force, and *R* and *T* represent the gas constant and the temperature, respectively. For simplicity, the particle velocity *ξ* and the fluid velocity ***u*** have been normalized by $\sqrt{3RT}$, giving a sound speed of $c_{s}=1/\sqrt{3}$ [53]. So, in the following simulations of isothermal continuum flow, the temperature *T* is constant with $c_{s}{}^{2}=RT=1/3$.

With the Taylor expansion at approximately zero particle velocity at a low Mach number, the Maxwellian equilibrium distribution function $f^{eq}$ is approximated as [1]

(4) $$f^{eq}=W_{\alpha }\rho \left[1+\frac{\xi_{\alpha }\cdot u}{c_{s}^{2}}+\frac{{\left(\xi_{\alpha }\cdot u\right)}^{2}}{2c_{s}^{4}}-\frac{{\left|u\right|}^{2}}{2c_{s}^{2}}\right],\;c_{s}^{2}=RT$$

In the widely used D2Q9 model [5], the velocity space $\left\{\xi_{\alpha }\right\}$ and the corresponding weights $\left\{W_{\alpha }\right\}$ are given as

(5) $$\xi_{\alpha }=\left\{\begin{array}{cc} \left(0,0\right) & \alpha =0,\\ c\left(\cos\left[\left(\alpha -1\right)\pi /2\right],\sin\left[\left(\alpha -1\right)\pi /2\right]\right)\;,c=\sqrt{3RT} & \alpha =1,2,3,4,\\ \sqrt{2}c\left(\cos\left[\left(2\alpha -9\right)\pi /4\right],\sin\left[\left(2\alpha -9\right)\pi /4\right]\right)\;,c=\sqrt{3RT} & \alpha =5,6,7,8, \end{array}\right.$$

(6) $$W_{\alpha }=\left\{\begin{array}{ll} \frac{4}{9},\; & \alpha =0\\ \frac{1}{9},\; & \alpha =1,2,3,4\\ \frac{1}{36},\; & \alpha =5,6,7,8 \end{array}\right.$$

The computational domain is divided into the cells *V_i,j_* = Δ*x_i_*Δ*y_j_* centered at (*x_i_*, *y_j_*) in the DUGKS with the D2Q9 model. As a finite volume scheme, the cell-averaged values of the distribution function and the force term are introduced, denoted as $f^{n}$ and $S^{n}$,

(7) $$\begin{array}{l} f^{n}=\frac{1}{\left|V_{i,j}\right|}\int_{V_{i,j}}f\left(\xi_{\alpha },x,t_{n}\right)dV,\\ S^{n}=\frac{1}{\left|V_{i,j}\right|}\int_{V_{i,j}}S\left(\xi_{\alpha },x,t_{n}\right)dV. \end{array}$$

Integrating Equation (1) into each cell *V_i,j_* from time *t_n_ = n*Δ*t* to time *t_n_*_+1_  = (*n* + 1)Δ*t*, and using the midpoint rule and trapezoidal rule, the evolution equation of the DUGKS is advanced from *t_n_* to *t_n+_*_1_ as,

(8) $$f^{n+1}-f^{n}=-\frac{\Delta t}{\left|V_{i,j}\right|}F^{n+1/2}+\frac{\Delta t}{2}\left[\Omega^{n}+\Omega^{n+1}\right]+\frac{\Delta t}{2}\left[S^{n}+S^{n+1}\right]$$

The scalar variable $F^{n+1/2}$ represents the flux across the cell interface, which is computed as

(9) $$F^{n+1/2}=\int_{\partial V_{i,j}}\left(\xi_{\alpha }\cdot n\right)f(\xi_{\alpha },x_{b},t_{n+1/2})dS$$

where *f* (***ξ****_α_*, ***x**_b_*, *t_n_*_+1/2_) represents the distribution at the cell interface ***x****_b_* at the time *t_n_*_+1/2_ = *t_n_* + *h* (*h* = Δ*t*/2), and ***n*** is the outward unit vector normal to the surface ∂*V_i_*_,*j*_.

For the purpose of eliminating the simplicity, new distribution functions are introduced:

(10) $$\tilde{f}\equiv f-\frac{\Delta t}{2}\Omega -\frac{\Delta t}{2}S,\;{\tilde{f}}^{+}\equiv f+\frac{\Delta t}{2}\Omega +\frac{\Delta t}{2}S$$

The collision term can be expanded in the BGK collision model, and Equation (10) can be converted to the following equations:

(11) $$f=\frac{2\tau }{2\tau +\Delta t}\tilde{f}+\frac{\Delta t}{2\tau +\Delta t}f^{eq}+\frac{\tau \Delta t}{2\tau +\Delta t}S\;,$$

(12) $${\tilde{f}}^{+}=\frac{2\tau -\Delta t}{2\tau +\Delta t}\tilde{f}+\frac{2\Delta t}{2\tau +\Delta t}f^{eq}+\frac{2\tau \Delta t}{2\tau +\Delta t}S\;.$$

The evolution equation of the DUGKS is simplified as

(13) $${\tilde{f}}_{}^{n+1}={\tilde{f}}_{}^{+,n}-\frac{\Delta t}{\left|V_{i,j}\right|}F_{}^{n+1/2}$$

All other forms of the distribution function can be expressed in terms of $\tilde{f}$ in the DUGKS. So, we focus on the distribution function $\tilde{f}$.

As can be seen from Equation (13), the critical step is to evaluate the interface flux $F_{}^{n+1/2}$.

For DUGKS with higher-order accuracy, more intermediate time steps can be selected, for example, the flux at the cell interface at *t*^∗^ = *t*_n_ + ∆*t*/6 and *t*^∗∗^ = *t*_n_ + 3∆*t*/4 need calculating [54]. Considering the easy implementation and fast computation in the present study, we select the intermediate time step $t_{n}+\Delta t/2$ in the present study. Equation (1) is integrated within a half time step (*h* = Δ*t*/2) along the characteristic line using the trapezoidal rule to treat the collision and force terms, and we have

(14) $$\begin{array}{l} f\left(\xi_{\alpha },x_{b},t_{n+1/2}\right)-f\left(\xi_{\alpha },x_{b}-h\xi_{\alpha },t_{n}\right)=\\ \frac{h}{2}\left[\Omega \left(\xi_{\alpha },x_{b},t_{n+1/2}\right)+\Omega \left(\xi_{\alpha },x_{b}-h\xi_{\alpha },t_{n}\right)\right]+\frac{h}{2}\left[S\left(\xi_{\alpha },x_{b},t_{n+1/2}\right)+S\left(\xi_{\alpha },x_{b}-h\xi_{\alpha },t_{n}\right)\right] \end{array}$$

where ***x****_b_* at the cell interface denotes an end point along the characteristic line.

Similar to the above discussion, new distribution functions are introduced:

(15) $$\bar{f}=f-\frac{h}{2}\Omega -\frac{h}{2}S=\frac{2\tau +h}{2\tau }f-\frac{h}{2\tau }f^{eq}-\frac{h}{2}S$$

(16) $${\bar{f}}^{+}=\frac{2\tau -h}{2\tau +h}\bar{f}+\frac{2h}{2\tau +h}f^{eq}+\frac{2\tau h}{2\tau +h}S.$$

With new distribution functions, Equation (14) is simplified as

(17) $$\bar{f}\left(\xi_{\alpha },x_{b},t_{n+1/2}\right)={\bar{f}}^{+}\left(\xi_{\alpha },x_{b}-h\xi_{\alpha },t_{n}\right)$$

With the Taylor expansion [1], the right term of Equation (17) can be approximated as

(18) $${\bar{f}}^{+}\left(\xi_{\alpha },x_{b}-h\xi_{\alpha },t_{n}\right)={\bar{f}}^{+}\left(\xi_{\alpha },x_{b},t_{n}\right)-h\xi_{\alpha }\cdot \nabla {\bar{f}}^{+}\left(\xi_{\alpha },x_{b},t_{n}\right)$$

where ${\bar{f}}_{}^{+}\left(\xi_{\alpha },x_{b},t_{n}\right)$ and its gradients $\nabla {\bar{f}}_{}^{+}\left(\xi_{\alpha },x_{b},t_{n}\right)$ can be approximated by linear interpolations. For the uniform mesh in the x-direction, e.g.,

(19) $$\begin{array}{l} \nabla {\bar{f}}^{+}\left(\xi_{\alpha },x_{b},t_{n}\right)\approx \frac{{\bar{f}}^{+}\left(\xi_{\alpha },x_{i+1},t_{n}\right)-{\bar{f}}^{+}\left(\xi_{\alpha },x_{i},t_{n}\right)}{(\Delta x_{i+1}+\Delta x_{i})/2},\\ {\bar{f}}^{+}\left(\xi_{\alpha },x_{b},t_{n}\right)\approx {\bar{f}}^{+}\left(\xi_{\alpha },x_{i},t_{n}\right)+\nabla {\bar{f}}^{+}\left(\xi_{\alpha },x_{b},t_{n}\right)\frac{\Delta x_{i}}{2}. \end{array}$$

The distribution function ${\bar{f}}^{+}$(***ξ****_α_*, ***x***, *t_n_*) at the cell center can be calculated as

(20) $${\bar{f}}^{+}=\frac{2\tau -h}{2\tau +\Delta t}\tilde{f}+\frac{3h}{2\tau +\Delta t}f^{eq}+\frac{3\tau h}{2\tau +\Delta t}S$$

Then, $\bar{f}(\xi_{\alpha },x_{b},t_{n+1/2})$ can be obtained by using Equations (17) and (18).

Similarly, the original distribution function $f(\xi_{\alpha },x_{b},t_{n+1/2})$ can be obtained from $\bar{f}(\xi_{\alpha },x_{b},t_{n+1/2})$:

$$f\left(\xi_{\alpha },x_{b},t_{n+1/2}\right)=\frac{2\tau }{2\tau +h}\bar{f}\left(\xi_{\alpha },x_{b},t_{n+1/2}\right)+\frac{h}{2\tau +h}f^{eq}\left(\xi_{\alpha },x_{b},t_{n+1/2}\right)$$

The equilibrium function $f^{eq}(\xi_{\alpha },x_{b},t_{n+1/2})$ at the cell interface can be determined by the conserved variables. Based on the conservation of mass and momentum, the density and velocity at the cell interface center can be given as

(21) $${\left.\rho_{n+1/2}\right|}_{x_{b}}=\sum_{\alpha }\bar{f}\left(\xi_{\alpha },x_{b},t_{n+1/2}\right),\;{\left.{\left(\rho u\right)}_{n+1/2}\right|}_{x_{b}}=\sum_{\alpha }\xi_{\alpha }\bar{f}\left(\xi_{\alpha },x_{b},t_{n+1/2}\right)+0.5\rho ah$$

Finally, the flux $F^{n+1/2}$ is obtained by Equation (9) after $f\left(\xi_{\alpha },x_{b},t_{n+1/2}\right)$ is determined by Equation (15). Then, the tracked distribution function $\tilde{f}$ can be updated to the next time step by Equation (13).

Particularly, the following equation will be used in the DUGKS [3],

(22) $${\tilde{f}}_{}^{+}=\frac{4}{3}{\bar{f}}_{}^{+}-\frac{1}{3}\tilde{f}$$

The density *ρ* and velocity ***u*** at the cell center can be calculated from $\tilde{f}$:

(23) $$\rho =\sum_{\alpha }\tilde{f}(\xi_{\alpha },x,t_{n}),\;\rho u=\sum_{\alpha }\xi_{\alpha }\tilde{f}(\xi_{\alpha },x,t_{n})+0.5\rho a\Delta t$$

In the present DUGKS, the relaxation time *τ* is computed from *τ = ν*/*RT*, where *ν* is the kinematic viscosity. The time step Δ*t* is independent of the relaxation time *τ* for all flow regimes, and it is determined by the Courant–Friedrichs–Lewy (CFL) condition [55]:

(24) $$\Delta t=\chi \frac{\Delta x}{C}$$

where *χ* is the CFL number, Δ*x* is the minimum grid spacing, and *C* is the maximum discrete velocity. We set *C* to be $\sqrt{2}$ in the present work.

### 2.2. Original Schemes of No-Slip Boundary Conditions

The original schemes of BB, NEBB, and Moment-based boundary conditions are introduced, and they are analyzed theoretically from the view of the moment.

The boundary conditions are applied to solve the unknown distributions that have velocities pointing into the fluid domain. So, we need to find the unknown distributions.

#### 2.2.1. Moment-Based Scheme

In brief, the Moment-based scheme states that the unknown distribution functions at the boundary can be determined by the linearly independent moments. For the D2Q9 model, there are nine discrete velocity moments, including the 6 hydrodynamic moments (the density *ρ*, momentum *ρu,* and momentum flux Π) and 3 non-hydrodynamic moments (*Q_xxy_*, *Q_xyy_,* and *S_xxyy_*) [40]:

(25) $$\begin{array}{l} \rho =\sum_{\alpha =0}^{8}f_{\alpha },\;\Pi_{x}=\rho u_{x}=\sum_{\alpha =0}^{8}f_{\alpha }\xi_{\alpha x},\;\Pi_{y}=\rho u_{y}=\sum_{\alpha =0}^{8}f_{\alpha }\xi_{\alpha y},\\ \Pi_{xx}=\sum_{\alpha =0}^{8}f_{\alpha }\xi_{\alpha x}\xi_{\alpha x},\;\Pi_{yy}=\sum_{\alpha =0}^{8}f_{\alpha }\xi_{\alpha y}\xi_{\alpha y},\;\Pi_{xy}=\sum_{\alpha =0}^{8}f_{\alpha }\xi_{\alpha x}\xi_{\alpha y}, \end{array}$$

(26) $$Q_{xxy}=\sum_{\alpha =0}^{8}f_{\alpha }\xi_{\alpha y}\xi_{\alpha x}^{2},\;Q_{xyy}=\sum_{\alpha =0}^{8}f_{\alpha }\xi_{\alpha x}\xi_{\alpha y}^{2},\;S_{xxyy}=\sum_{\alpha =0}^{8}f_{\alpha }\xi_{\alpha x}^{2}\xi_{\alpha y}^{2}.$$

For the no-slip wall boundary, we pick three linearly independent moments (momentum Π*_x_*, Π*_y_,* and momentum flux Π*_xx_*), and impose constraints upon them, i.e., *u_x_* = *u_wx_* (const), *u_y_* = 0, ∂*_x_u_x_* = 0 for the south boundary (*u_wy_* = 0). Then, by Chapman–Enskog, Π*_xx_* = Π*_xx_*^(0)^ + *τ*Π*_xx_*^(1)^ = Π*_xx_*^(0)^ = *c_s_*^2^*ρ* + *ρu_x_*^2^, because Π*_xx_*^(1)^ ≈ −2*c_s_*^2^*ρ*∂*_x_u_x_* = 0. We obtain the following three conditions for three unknowns:

(27) $$\Pi_{x}=\rho u_{wx},\;\Pi_{y}=0,\;\Pi_{xx}=\Pi_{xx}^{\left(0\right)}=\rho /3+\rho u_{wx}^{2}$$

For the south boundary (*u_wy_* = 0), solving these equations yields

(28) $$\begin{array}{l} f_{2}=f_{1}+f_{3}+f_{4}+2(f_{7}+f_{8})-\rho /3-\rho u_{wx}^{2}\\ f_{5}=\rho /6-f_{1}-f_{8}+0.5\rho u_{wx}^{2}+0.5\rho u_{wx}\\ f_{6}=\rho /6-f_{3}-f_{7}+0.5\rho u_{wx}^{2}-0.5\rho u_{wx}\\ \rho =f_{0}+f_{1}+f_{3}+2(f_{4}+f_{7}+f_{8}) \end{array}$$

#### 2.2.2. Non-Equilibrium Bounce Back Scheme

For the south boundary (*u_wy_* = 0), the Non-equilibrium Bounce-Back (NEBB) scheme can be expressed as [38]:

(29) $$\begin{array}{l} f_{2}=f_{4}\\ f_{5}=f_{7}-0.5(f_{1}-f_{3})+0.5\rho u_{wx}\\ f_{6}=f_{8}+0.5(f_{1}-f_{3})-0.5\rho u_{wx}\\ \rho =f_{0}+f_{1}+f_{3}+2(f_{4}+f_{7}+f_{8}) \end{array}$$

where *u_wx_* denotes the wall velocity in the *x*-direction.

It is found that the calculation of the density in Equation (28) is the same as that in Equation (29) when *u_wy_* = 0. Then, from a fresh perspective based on the moment, it is found that the conditions of the NEBB scheme are

(30) $$\Pi_{x}=\rho u_{wx},\;\Pi_{y}=0,\;Q_{xxy}=\rho u_{wy}/3=0.$$

That is, for the NEBB scheme, three linearly independent moments are picked for the south boundary: Momentum Π*_x_*, Π*_y_*, and momentum flux *Q_xxy_*. Π*_x_* and Π*_y_* are useful in defining the no-slip condition, but the condition on a component of the third-order moment *Q_xxy_* seems somewhat arbitrary.

#### 2.2.3. Bounce-Back Scheme

The Bounce-Back (BB) scheme assumes that a particle just reverses its velocity after hitting the wall. The unknown distribution functions *f* (***x****_w_*, ***ξ****_α_*, *t_n+_*_1/2_) for particles leaving the wall are determined as [1],

(31) $$f\left(x_{w},\xi_{\alpha },t_{n+1/2}\right)=f\left(x_{w},-\xi_{\alpha },t_{n+1/2}\right)+2\rho_{w}W_{\alpha }\frac{\xi_{\alpha }\cdot u_{w}}{RT},\left(\xi_{\alpha }\cdot n>0\right)$$

(32) $$RT=c_{s}^{2}$$

where ***u****_w_* denotes the wall velocity and *ρ_w_* denotes the density at the wall boundary, which can be approximated well by the constant average density for nearly incompressible flows [1].

For the south boundary (the wall velocity at y-direction *u_wy_* = 0), the unknown original distribution functions $f_{2}$, $f_{5}$, and $f_{6}$ are given as,

(33) $$\begin{array}{l} f_{2}=f_{4}\\ f_{5}=f_{7}+\rho_{w}u_{wx}/6\\ f_{6}=f_{8}-\rho_{w}u_{wx}/6 \end{array}$$

where *u_wx_* denotes the wall velocity in the *x*-direction.

For the BB scheme, three linearly independent moments are picked for the south boundary: Π*_y_*, *Q_xyy_,* and *Q_xxy_*, leading to the conditions on a moment basis,

(34) $$\Pi_{y}=0,\;Q_{xyy}=\rho u_{wx}/3,\;Q_{xxy}=0.$$

Two components of the third-order moment *Q_xyy_* and *Q_xxy_* result in the numerical slip error.

### 2.3. Novel Schemes of No-Slip Boundary Conditions

In the DUGKS, new transformed distribution functions are introduced to evaluate the interface flux. So, the original scheme of boundary conditions should be transformed. The original distribution functions f(***x****_w_*, ***ξ****_α_*, *t* + *h*) are obtained after the transformed distribution functions $\bar{f}$(***x****_w_*, ***ξ****_α_*, *t* + *h*) are calculated. So, the implementation of the boundary condition shifts from the original f(***x****_w_*, ***ξ****_α_*, *t* + *h*) to the transformed $\bar{f}$(***x****_w_*, ***ξ****_α_*, *t* + *h*), which may improve the numerical performance. It is noted that the particle streaming and collision are fully coupled in the DUGKS.

**Figure 1:**
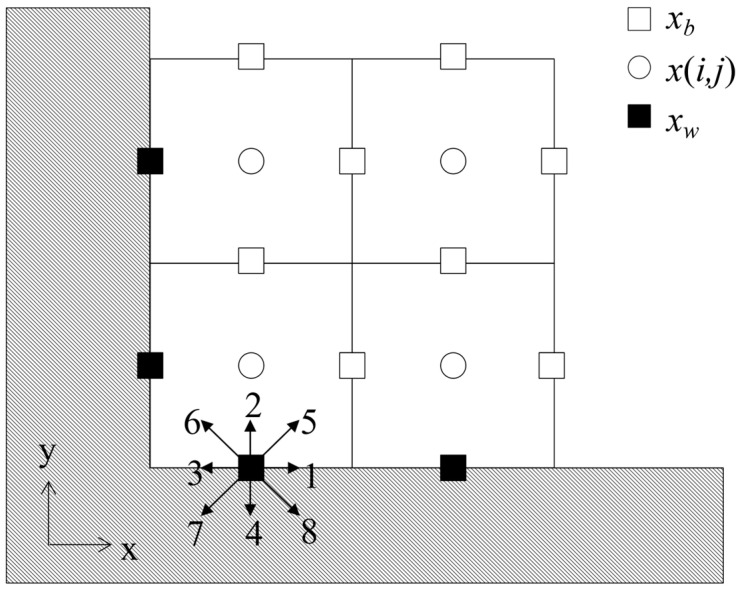
Schematic of cell geometry near the boundary.

As shown in Figure 1, in the present work, the boundary is located at a cell interface, and we treat the unknown distribution functions $\bar{f}$(***x****_w_*, ***ξ****_α_*, *t* + *h*) at the cell interface center ***x****_w_*. Owing to new distribution functions and force terms in the DUGKS, we should convert the original boundary conditions into a new format. The boundary conditions will be translated into constraints on $\bar{f}$(***x****_w_*, ***ξ****_α_*, *t* + *h*). It should noted that more details about derivations for the novel schemes are shown in Appendix A.

Considering Equation (21), we can obtain

(35) $$\Pi_{x}=\rho u_{x}={\bar{f}}_{1}-{\bar{f}}_{3}+{\bar{f}}_{5}-{\bar{f}}_{6}-{\bar{f}}_{7}+{\bar{f}}_{8}+0.5\rho a_{x}h$$

(36) $$\Pi_{y}=\rho u_{y}={\bar{f}}_{2}-{\bar{f}}_{4}+{\bar{f}}_{5}+{\bar{f}}_{6}-{\bar{f}}_{7}-{\bar{f}}_{8}+0.5\rho a_{y}h$$

With Equations (27), (35), and (36), the Moment-based scheme (Equation (28)) is rewritten as

(37) $$\begin{array}{l} {\bar{f}}_{2}={\bar{f}}_{1}+{\bar{f}}_{3}+{\bar{f}}_{4}+2({\bar{f}}_{7}+{\bar{f}}_{8})-\rho /3-\rho u_{wx}^{2}-0.5\rho a_{y}h\\ {\bar{f}}_{5}=\rho /6-{\bar{f}}_{1}-{\bar{f}}_{8}+0.5\rho u_{wx}^{2}+0.5\rho u_{wx}-0.25\rho a_{x}h\\ {\bar{f}}_{6}=\rho /6-{\bar{f}}_{3}-{\bar{f}}_{7}+0.5\rho u_{wx}^{2}-0.5\rho u_{wx}+0.25\rho a_{x}h\\ \rho =[{\bar{f}}_{0}+{\bar{f}}_{1}+{\bar{f}}_{3}+2({\bar{f}}_{4}+{\bar{f}}_{7}+{\bar{f}}_{8})]/(1+0.5a_{y}h) \end{array}$$

In the Couette flow and lid-driven cavity flow, the top wall moves with the constant velocity *U*_0_ (*a_x_* = *a_y_* = 0, *u_wx_* = *U*_0_, *u_wy_* = 0, ∂*_x_u_wx_* = 0), and the unknowns at the north boundary can be written as

(38) $$\begin{array}{l} {\bar{f}}_{4}={\bar{f}}_{1}+{\bar{f}}_{2}+{\bar{f}}_{3}+2({\bar{f}}_{5}+{\bar{f}}_{6})-\rho /3-\rho U_{0}{}^{2}\\ {\bar{f}}_{7}=\rho /6-{\bar{f}}_{3}-{\bar{f}}_{6}+0.5\rho U_{0}{}^{2}-0.5\rho U_{0}\\ {\bar{f}}_{8}=\rho /6-{\bar{f}}_{1}-{\bar{f}}_{5}+0.5\rho U_{0}{}^{2}+0.5\rho U_{0}\\ \rho ={\bar{f}}_{0}+{\bar{f}}_{1}+{\bar{f}}_{3}+2({\bar{f}}_{2}+{\bar{f}}_{5}+{\bar{f}}_{6}) \end{array}$$

With Equations (30), (35), and (36), the NEBB scheme (Equation (29)) is rewritten as

(39) $$\begin{array}{l} {\bar{f}}_{2}={\bar{f}}_{4}-0.5\rho a_{y}h\\ {\bar{f}}_{5}={\bar{f}}_{7}-({\bar{f}}_{1}-{\bar{f}}_{3})/2+0.5\rho u_{wx}-0.25\rho a_{x}h\\ {\bar{f}}_{6}={\bar{f}}_{8}+({\bar{f}}_{1}-{\bar{f}}_{3})/2-0.5\rho u_{wx}+0.25\rho a_{x}h\\ \rho =[{\bar{f}}_{0}+{\bar{f}}_{1}+{\bar{f}}_{3}+2({\bar{f}}_{4}+{\bar{f}}_{7}+{\bar{f}}_{8})]/(1+0.5a_{y}h) \end{array}$$

With Equations (34) and (36), the BB scheme (Equation (33)) is rewritten as

(40) $$\begin{array}{l} {\bar{f}}_{2}={\bar{f}}_{4}-0.5\rho a_{y}h\\ {\bar{f}}_{5}={\bar{f}}_{7}+\rho u_{wx}/6\\ {\bar{f}}_{6}={\bar{f}}_{8}-\rho u_{wx}/6\\ \rho =[{\bar{f}}_{0}+{\bar{f}}_{1}+{\bar{f}}_{3}+2({\bar{f}}_{4}+{\bar{f}}_{7}+{\bar{f}}_{8})]/(1+0.5a_{y}h) \end{array}$$

### 2.4. Analysis of Numerical Slip Errors of Novel Schemes

In the following, the numerical slip errors ($u_{\mathrm{error}}=u_{x}-u_{wx}$) of the novel schemes of no-slip boundary conditions are theoretically analyzed.

Inspired by Guo et al. [56], He et al. [57], Wang et al. [58], and Yang et al. [59], the unidirectional steady flow is adopted to analyze the numerical error of boundary conditions. The assumptions in the unidirectional steady flow are written as

(41) $$u_{y}=0,\;a_{y}=0,\;\partial \phi /\partial x=0,\;\partial \phi /\partial t=0,\;\rho =\mathrm{const},$$

where ϕ denotes an arbitrary flow variable. *a_x_* and *a_y_* denote the component of the acceleration ***a*** in the *x*-direction and *y*-direction, respectively. Considering Equations (14) and (35), we can obtain

(42) $$f_{1}=\tau a_{x}(c-u_{x})f_{1}^{eq}/RT+f_{1}^{eq},f_{3}=\tau a_{x}(-c-u_{x})f_{3}^{eq}/RT+f_{3}^{eq}$$

With the Maxwellian equilibrium distribution function known, as shown in Equation (4), we can obtain

(43) $$f_{1}-f_{3}=2\rho (\tau a_{x}+u_{x})/3$$

Yang et al. [39] treated the unknown distribution functions *f*(***x****_w_*, ***ξ****_α_*, *t* + *h*) at the cell interface ***x****_w_*, and the numerical slip error of the BB scheme and NEBB scheme generated in the DUGKS can be written as

(44) $${u^{\prime }}_{error,\mathrm{BB}}=2\tau a_{x},\;{u^{\prime }}_{error,\mathrm{NEBB}}=0.$$

However, in the present work, we treat the unknown distribution functions $\bar{f}$(***x****_w_*, ***ξ****_α_*, *t* + *h*) at the cell interface center ***x****_w_*.

For the Moment-based scheme, we substitute Equation (37) into Equation (35), and obtain

(45) $$u_{\mathrm{error},\mathrm{Moment}}=u_{x}-u_{wx}=0$$

For the NEBB scheme, we substitute Equation (39) into Equation (35), and obtain

(46) $$u_{\mathrm{error},\mathrm{NEBB}}=u_{x}-u_{wx}=0$$

For the BB scheme, we substitute Equation (40) into Equation (35), and obtain

(47) $$\rho u_{x}={\bar{f}}_{1}-{\bar{f}}_{3}+\rho u_{wx}/3+0.5\rho a_{x}h$$

With Equations (15) and (43), we can obtain

(48) $${\bar{f}}_{1}-{\bar{f}}_{3}=\frac{2\tau +h}{3}\rho a_{x}+\frac{2}{3}\rho u_{x}-ha_{x}u_{x}\left(1-u_{x}\right)$$

It is found that the numerical slip error of the BB scheme in the present work is also zero ($u_{\mathrm{error},\mathrm{BB}}=u_{x}-u_{wx}=0$) when the acceleration *a_x_* is equal to 0.

As shown in Equation (34), the only hydrodynamic condition is that the flow rate through the wall is zero in the BB scheme, and the velocity along the wall is undefined. The BB scheme does not impose a boundary condition on the tangential velocity at the wall, resulting in the numerical slip velocity when the external force term exists. Both the NEBB scheme and Moment-based scheme select momentum Π*_x_* and Π*_y_*, which can satisfy the no-slip condition without generating numerical slip error. So, momentum Π*_x_* and Π*_y_* are useful in defining the no-slip condition. It seems sensible to choose the hydrodynamic moments instead of the non-hydrodynamic ones that do not appear in these equations of motion [40].

### 2.5. Explore Extending Present Schemes to Curved Boundaries

To extend the current boundary treatment to curved boundaries, this work also tries to introduce the methods from the LBM into the DUGKS, including the link method (Ladd [60,61]), interpolated methods (Bouzidi et al. [62], Yu et al. [63]), and single-node schemes (Zhao&Yong [64], Tao et al. [65], Zhao et al. [66], Chen et al. [67]). Although the LBM and DUGKS share a common kinetic origin, we need to be aware of distinctive features in the LBM and DUGKS. It should be noted that the present boundary conditions treat the unknown transformed distribution functions $\bar{f}$ at the cell interface in the DUGKS.

#### 2.5.1. Link Method

**Figure 2:**
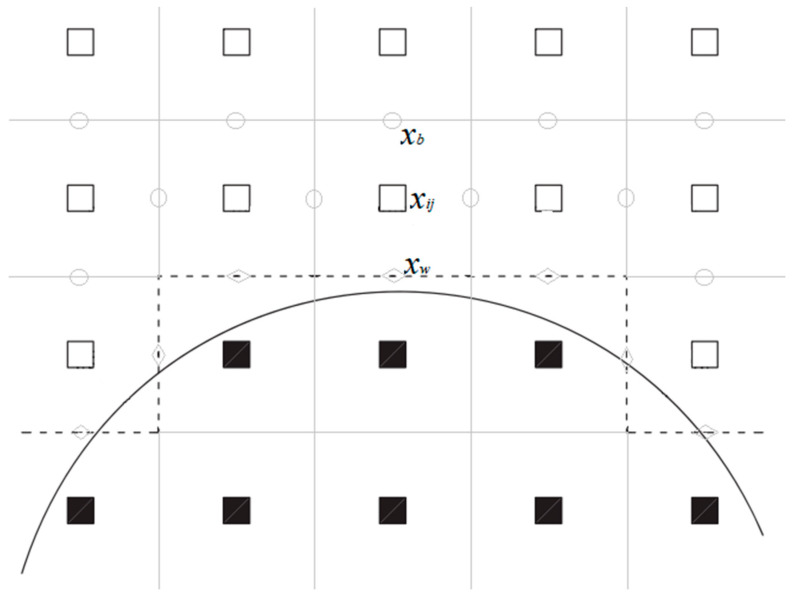
Link method approximates curved boundary to zigzag one. The white square means fluid node ***x****_ij_*, black square means solid node, solid line means real curved boundary, and dashed line means numerical boundary. Gray circle denotes the cell interface center ***x****_b_*. Gray diamond denotes the numerical boundary node ***x****_w_*, which is located at the cell interface center on the numerical boundary.

As shown in Figure 2, the link method approximates the curved solid boundary to a stair-like zigzag line (dash line), which is set in the center of the solid and fluid nodes (i.e., the cell interface). So, it is quite convenient for the DUGKS to apply the link method to treat the curved boundaries by treating the unknown transformed distribution functions $\bar{f}$(***x****_w_*, ***ξ****_α_*, *t* + *h*) at the cell interface center ***x****_w_* and at time *t_n_*_+1/2_ = *t_n_* + 0.5Δ*t* (*h* = 0.5Δ*t*). If the grid mesh is fine enough, it is easy and efficient to treat the curved boundaries by adopting the current boundary treatment (as shown in Section 2.3) directly.

#### 2.5.2. Interpolated Method

In actuality, the link method makes the smooth curved boundary into the rough boundary. To improve the curved boundary conditions, some interpolated methods are developed. It should be noted that strategies to design interpolated schemes are not unique. For example, in the LBM, two representative interpolated bounce-back (IBB) schemes are the conditional scheme proposed by Bouzidi et al. [62] and the unified scheme by Yu et al. [63]. It is noted that the interpolated methods are based on the bounce-back scheme in the LBM. Similarly, the present BB scheme for the DUGKS can be applied to the interpolated methods. However, it needs more implementation effort or other strategies to extend the present NEBB scheme and Moment-based scheme to curved boundaries.

##### Conditional Scheme

Inspired by the conditional scheme [62], the IBB scheme in the DUGKS is proposed by using linear (first-order treatment) or quadratic (second-order treatment) interpolation formulas involving values at two or three nodes. The conditional scheme uses separate treatments for *q* < 0.5 and *q* ≥ 0.5 according to the scaled distance.

**Figure 3:**
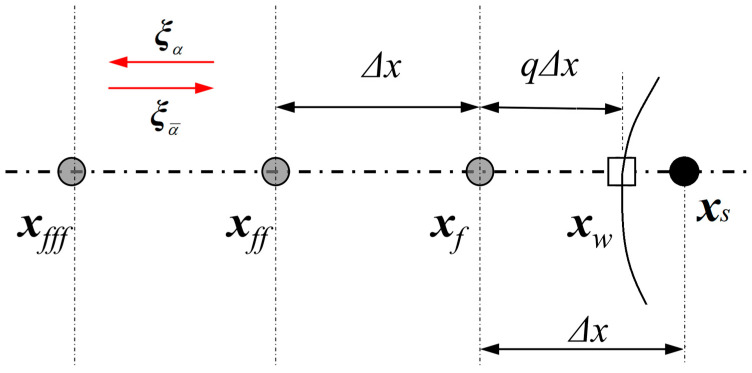
Sketch of a curved boundary located between two lattice nodes arbitrarily. Grey circles represent the fluid nodes (i.e., ***x****_f_*, ***x****_ff_*, ***x****_fff_*), black circle represents the solid node (***x****_s_*), and square box represents the intersection (***x****_w_*) of the boundary and the grid line. ***ξ****_α_* defines the lattice velocity of the particle, which travels from ***x****_f_* to ***x****_ff_* and $\xi_{\bar{\alpha }}$ denotes the opposite direction of ***ξ****_α_* ($\xi_{\bar{\alpha }}$ = $-\xi_{\alpha }$). *q* (*q* = |***x****_w_* − ***x****_f_*|/|***x****_s_* − ***x****_f_*|) denotes the scaled distance.

Taking the boundary configuration in Figure 3 as an example, we use quadratic interpolation to obtain,

(49) $$\begin{array}{l} \bar{f}\left(x_{f},\xi_{\alpha },t_{n+1/2}\right)=q(2q+1)\bar{f}\left(x_{f},\xi_{\bar{\alpha }},t_{n-1/2}\right)+(1+2q)(1-2q)\bar{f}\left(x_{ff},\xi_{\bar{\alpha }},t_{n-1/2}\right)\\ -q(1-2q)\bar{f}\left(x_{fff},\xi_{\bar{\alpha }},t_{n-1/2}\right)+2\rho_{w}W_{\alpha }\frac{\xi_{\alpha }\cdot u_{w}}{c_{s}^{2}},\;\;q<0.5, \end{array}$$

(50) $$\begin{array}{l} \bar{f}\left(x_{f},\xi_{\alpha },t_{n+1/2}\right)=\frac{1}{q(2q+1)}\left[\bar{f}\left(x_{f},\xi_{\bar{\alpha }},t_{n-1/2}\right)+2\rho_{w}W_{\alpha }\frac{\xi_{\alpha }\cdot u_{w}}{c_{s}^{2}}\right]\\ +\frac{2q-1}{q}\bar{f}\left(x_{ff},\xi_{\alpha },t_{n+1/2}\right)-\frac{2q-1}{2q+1}\bar{f}\left(x_{fff},\xi_{\alpha },t_{n+1/2}\right),\;\;q\geq 0.5. \end{array}$$

When *q* = ½, the conditional interpolation formulas are reduced to the “bounce-back” scheme. Since a distribution function travels precisely one grid spacing from *t* to *t* + ∆*t*, particles that start from ***x****_f_* can end precisely at the same location only when ***x****_f_* is half a grid spacing from the wall.

##### Unified Scheme

Inspired by the unified scheme [63], the present work treats the curved boundaries in three main steps:

➀ A virtual distribution function $\bar{f}$(***x****_w_*, $\xi_{\bar{\alpha }}$, *t* + *h*) is interpolated from the distribution functions at ***x****_f_*, ***x****_ff_* and ***x****_fff_*. We use quadratic interpolation to obtain,
  (51) $$\begin{array}{l} \bar{f}(x_{w},\xi_{\bar{\alpha }},t_{n+1/2})=\frac{q(q+1)}{2}\bar{f}(x_{f},\xi_{\bar{\alpha }},t_{n-1/2})+\\ \left(1+q)(1-q)\bar{f}(x_{ff},\xi_{\bar{\alpha }},t_{n-1/2})-\frac{q(1-q)}{2}\bar{f}(x_{fff},\xi_{\bar{\alpha }},t_{n-1/2}\right) \end{array}$$
➁ According to the current boundary treatment, the unknown distribution function $\bar{f}$(***x****_w_*, $\xi_{\alpha }$, *t* + *h*) is obtained by using the known distribution function $\bar{f}$(***x****_w_*, $\xi_{\bar{\alpha }}$, *t* + *h*). All the present BB, NEBB, and Moment-based schemes (as shown in Section 2.3) are alternatives to calculate $\bar{f}$(***x****_w_*, $\xi_{\alpha }$, *t* + *h*).
➂ At last, we need to calculate the known distribution function $\bar{f}$(***x****_f_*, $\xi_{\alpha }$, *t* + *h*), which is interpolated from $\bar{f}$(***x****_w_*, $\xi_{\alpha }$, *t* + *h*), $\bar{f}$(***x****_ff_*, $\xi_{\alpha }$, *t* + *h*), $\bar{f}$(***x****_fff_*, $\xi_{\alpha }$, *t* + *h*).
  (52) $$\begin{array}{l} \bar{f}(x_{f},\xi_{\alpha },t_{n+1/2})=\frac{2}{\left(1+q)(2+q\right)}\bar{f}(x_{w},\xi_{\alpha },t_{n+1/2})+\\ \frac{2q}{1+q}\bar{f}(x_{ff},\xi_{\alpha },t_{n+1/2})-\frac{q}{2+q}\bar{f}(x_{fff},\xi_{\alpha },t_{n+1/2}) \end{array}$$

#### 2.5.3. Single-Node Scheme

The interpolated methods [62,63] improve the accuracy to be second-order. However, they involve at least two neighboring fluid nodes, e.g., ***x****_f_*, ***x****_ff_,* and ***x****_fff_*. It is inevitable to undermine the local computation property and further the parallel performance of LBM [68,69]. Moreover, the required number of fluid nodes may not be available for some cases, e.g., dense particle suspensions. Zhao and Yong (2017) [64] developed a single-node second-order bounce-back scheme for curved boundaries, which used pre- and post-collision density distributions to determine the unknown density distributions at the boundary. An alternative single-node second-order scheme was proposed by Tao et al. (2018) [65]. Zhao et al. (2020) [66] derived a general single-node second-order scheme for curved boundaries. However, the free parameter is limited within the range of [max(0, 2*q* − 1), 2*q*]. Later, it was reported that the free parameter *γ* can be arbitrarily chosen within the range of [0, 2*q*] in a general single-node second-order scheme proposed by Chen et al. (2021) [67], but *γ* should be normalized by the gird spacing ∆*x*. It should be noted that the single-node schemes [64,65,66,67] do not involve the present Moment-based scheme.

Inspired by Zhao and Yong (2017) [64], the present single-node second-order BB scheme in the DUGKS can be similarly expressed as,

(53) $$\bar{f}(x_{f},\xi_{\alpha },t_{n+1/2})=\frac{2q}{1+2q}\bar{f}(x_{f},\xi_{\alpha },t_{n-1/2})+\frac{1}{1+2q}\bar{f}(x_{f},\xi_{\bar{\alpha }},t_{n-1/2})+\frac{2}{1+2q}\rho_{0}W_{\alpha }\frac{\xi_{\alpha }\cdot u_{w}}{c_{s}^{2}}$$

where *ρ*_0_ represents the mean density.

Inspired by Tao et al. (2018) [65], the present single-node second-order NEBB scheme in the DUGKS can be similarly expressed as,

(54) $$\bar{f}(x_{f},\xi_{\alpha },t_{n+1/2})=\frac{1}{1+q}\bar{f}(x_{w},\xi_{\alpha },t_{n+1/2})+\frac{q}{1+q}\bar{f}(x_{ff},\xi_{\alpha },t_{n+1/2})$$

(55) $$\bar{f}(x_{ff},\xi_{\alpha },t_{n+1/2})=\bar{f}(x_{f},\xi_{\alpha },t_{n-1/2})$$

(56) $$\begin{array}{l} \bar{f}(x_{w},\xi_{\alpha },t_{n+1/2})={\bar{f}}^{eq}(x_{w},\xi_{\alpha },t_{n+1/2})+{\bar{f}}^{neq}(x_{w},\xi_{\alpha },t_{n+1/2})\\ ={\bar{f}}^{eq}(x_{w},\xi_{\alpha },t_{n+1/2})+{\bar{f}}^{neq}(x_{f},\xi_{\alpha },t_{n+1/2})\\ ={\bar{f}}^{eq}(x_{w},\xi_{\alpha },t_{n+1/2})+{\bar{f}}^{neq}(x_{f},\xi_{\bar{\alpha }},t_{n-1/2}), \end{array}$$

As shown in Equation (55), it is known that the distribution function at ***x****_ff_* streams directly from ***x****_f_*. The distribution function at ***x****_w_* is divided into two parts of equilibrium and non-equilibrium, which are represented by the superscripts *eq* and *neq*, respectively.

For the equilibrium part at ***x****_w_*, it can be determined by the known velocity and the approximate fluid density, according to the Maxwellian equilibrium distribution function (Equation (4)). ${\bar{f}}^{eq}(x_{w},\xi_{\alpha },t_{n+1/2})$ is approximated as

(57) $$\begin{array}{l} {\bar{f}}^{eq}(x_{w},\xi_{\alpha },t_{n+1/2})={\bar{f}}_{\alpha }^{eq}(u_{w}(t+h),\rho_{w}(t+h))\\ \approx {\bar{f}}_{\alpha }^{eq}(u_{w}(t+h),\rho_{f}(t+h))\approx {\bar{f}}_{\alpha }^{eq}(u_{w}(t+h),\rho_{f}(t-h)) \end{array}$$

It has been demonstrated that for low-speed flow, using *ρ_f_* (*t*) and *ρ_f_* (*t* + *h*) to approximate *ρ_f_* (*t* + *h*) and *ρ_w_* (*t* + *h*) have second- and third-order accuracies, respectively [70].

For the non-equilibrium part, it can be approximated and calculated by the non-equilibrium distribution function at *x_f_*, with the idea of non-equilibrium bounce back [38,71]. ${\bar{f}}^{neq}(x_{w},\xi_{\alpha },t_{n+1/2})$ is approximated as

(58) $${\bar{f}}^{neq}(x_{w},\xi_{\alpha },t_{n+1/2})\approx {\bar{f}}^{neq}(x_{f},\xi_{\alpha },t_{n+1/2})\approx {\bar{f}}^{neq}(x_{f},\xi_{\bar{\alpha }},t_{n-1/2}).$$

${\bar{f}}^{neq}(x_{w},\xi_{\alpha },t_{n+1/2})$ is obtained having at least first-order accuracy, which is enough for deriving a second-order construction of $\bar{f}(x_{w},\xi_{\alpha },t_{n+1/2})$ [65]. Hence, the present single-node second-order NEBB scheme is second-order accurate in space theoretically.

**Figure 4:**
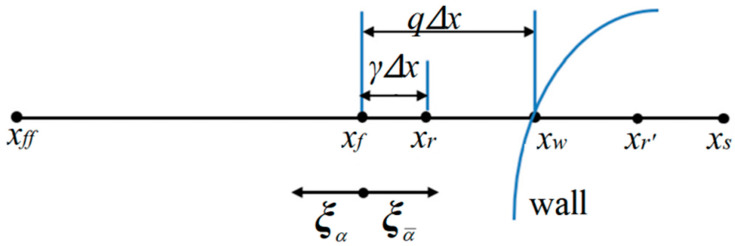
Sketch of a curved boundary with the auxiliary points *x_r_* and *x_r’_*(*x_r’_* − *x_w_* = *x_w_* − *x_r_*). *q* (*q* = |***x****_w_* − ***x****_f_*|/|***x****_s_* − ***x****_f_*|) denotes the scaled distance. *γ* (*γ* = |***x****_r_* − ***x****_f_*|/|***x****_s_* − ***x****_f_*|) is a non-negative free parameter.

Inspired by Chen et al. (2021) [67], taking the boundary configuration in Figure 4 as an example, a general single-node second-order scheme for the DUGKS is expressed as

(59) $$\begin{array}{l} \bar{f}(x_{f},\xi_{\alpha },t_{n+1/2})=\frac{\gamma }{1+\gamma }\bar{f}(x_{f},\xi_{\alpha },t_{n-1/2})+\\ \frac{1}{1+\gamma }\left((1+\gamma -2q)\bar{f}(x_{f},\xi_{\bar{\alpha }},t_{n-1/2})+(2q-\gamma )\bar{f}(x_{f},\xi_{\bar{\alpha }},t_{n+1/2})+2\rho_{0}W_{\alpha }\frac{\xi_{\alpha }\cdot u_{w}}{c_{s}^{2}}\right). \end{array}$$

The constraint 1 < *γ* ≤ 2*q* is applied to ensure that both 1 + *γ* − 2*q* and 2*q* − *γ* are non-negative.

In future work, the present schemes for curved boundaries in the DUGKS will be further validated and analyzed by theoretical analysis and numerical tests.

## 3. Numerical Tests

We perform the numerical tests of the Couette flow, the Poiseuille flow, the Lid-driven cavity flow, and the Rayleigh–Taylor instability to further assess the proposed scheme of boundary conditions for the DUGKS. In our simulations, the CFL number is set to be 0.95, and Re = *HU*_0_/*ν*.

The convergence criterion for attaining the steady-state solution is

(60) $$error=\sum_{i,j}\left|u_{ij}^{n}-u_{ij}^{n-1000}\right|/\sum_{i,j}\left|u_{ij}^{n}\right|\leq 10^{-6}$$

where $u_{ij}^{n}=u(x_{i},y_{j},n\Delta t)$ represents the velocity in the fluid domain.

To assess the accuracy, we measure the *L*_2_ errors of steady velocity fields,

(61) $$E(u)=\sqrt{\sum_{}(u-u_{}^{\prime }{)}^{2}}/\sqrt{\sum_{}(u_{}^{\prime }{)}^{2}}$$

where $u_{}^{\prime }$ is the analytical solution.

### 3.1. The Couette Flow

In the Couette flow, the top wall moves with the horizontal velocity *U*_0_ = 0.1, and the bottom wall is fixed. We apply the proposed scheme of boundary conditions to the top and bottom walls. The inlet and outlet adopt the periodic boundary condition. The gap between the top and bottom wall is set as *H* = 1. For the no-slip condition, the analytical solution of horizontal velocity in the Couette flow can be written as,

*u*(*y*)/*U*_0_ = *y*/*H*. (62)

It is noted that the convergence criterion for simulating the Couette flow follows Equation (60). To test the accuracy, the *L*_2_ errors of horizontal velocity along the vertical center line are measured at Re = 100, 1000, and 10,000. The results are shown in Table 1, Table 2 and Table 3. Under different meshes (N × N, N = 16, 32, 64, 96, 128), the time step is set as Δ*t* = 0.95√2/N = 0.08396893, 0.041984465, 0.020992233, 0.013994822, and 0.010496116, respectively.

**Figure 5:**
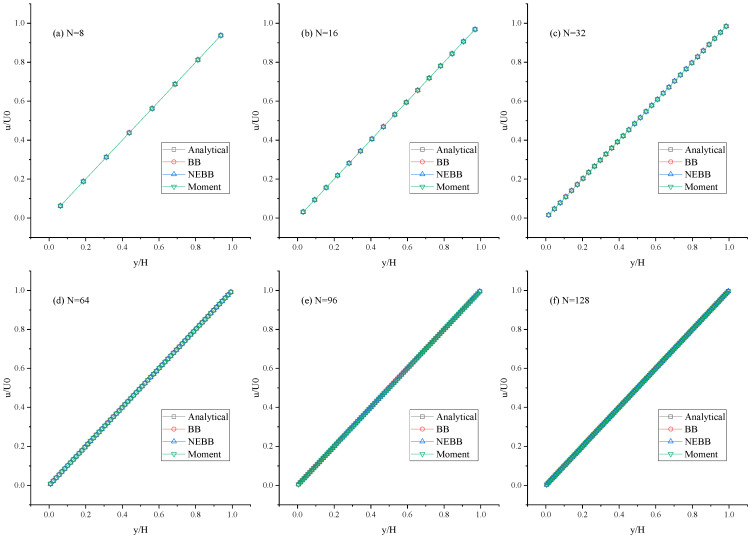
Horizontal velocity profiles of the Couette flow at Re = 100 under different meshes.

**Figure 6:**
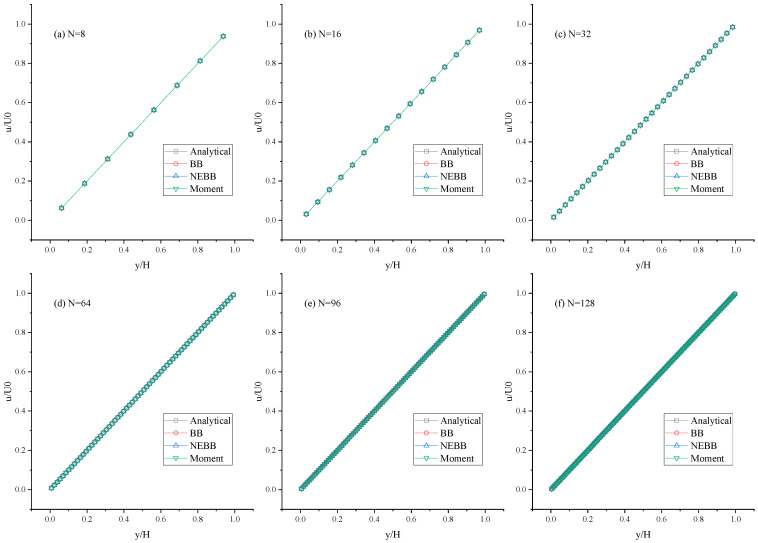
Horizontal velocity profiles of the Couette flow at Re = 1000 under different meshes.

**Figure 7:**
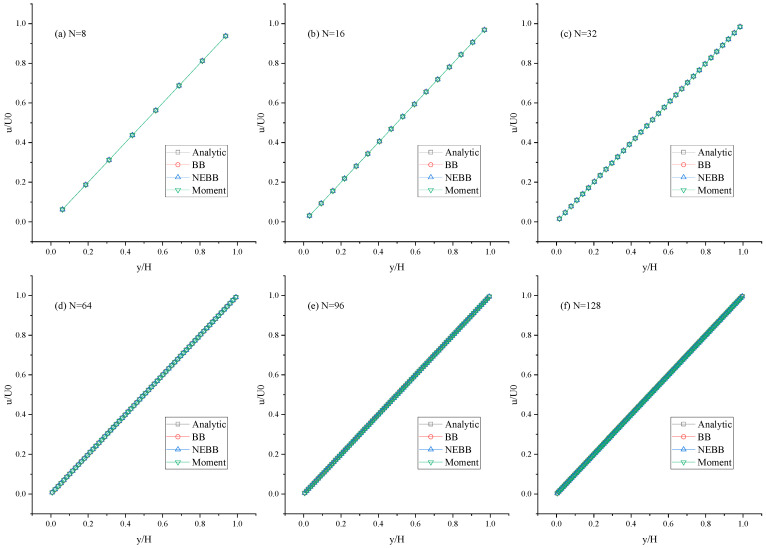
Horizontal velocity profiles of the Couette flow at Re = 10,000 under different meshes.

As shown in Figure 5, Figure 6 and Figure 7, the present results agree very well with the analytic solution. As shown in Table 1, Table 2 and Table 3, the numerical errors are almost negligible, even with a coarse mesh (N = 8). It is shown that the BB scheme, NEBB scheme, and Moment-based scheme can accurately simulate the Couette flow with the no-slip condition. As shown in Table 1, Table 2 and Table 3, the *L*_2_ errors in the Moment-based scheme are equal to those in the NEBB scheme, which are a little less than those in the BB scheme at Re = 100.

To test the stability of the proposed scheme of boundary conditions at high Re, some simulations are performed at Re = 10^5^, 10^6^, and 10^7^ with N = 16. The steps of reaching the steady state are shown in Table 4. It is found that both the NEBB scheme and thMoment-based scheme converge to the steady state faster than the BB scheme.

As shown in Table 5, the *L*_2_ error of the Moment-based scheme is equal to that of the NEBB scheme, and the *L*_2_ errors of the Moment-based scheme and the NEBB scheme are a little less than those in the BB scheme. The *L*_2_ errors are approximately 0.23%, 2.25%, and 18.6% at Re = 10^5^, 10^6^, and 10^7^, respectively. It is shown that the schemes can predict acceptable results for the simulation of the Couette flow at high Re.

### 3.2. The Lid-Driven Cavity Flow

In the lid-driven cavity flow, the top wall moves with the horizontal velocity *U*_0_ = 0.1. The bottom wall and left- and right-side walls are fixed. The walls adopt the present boundary conditions. The cavity length *L* is set to be 1. It is noted that the convergence criterion for simulating the lid-driven flow follows Equation (60).

**Figure 8:**
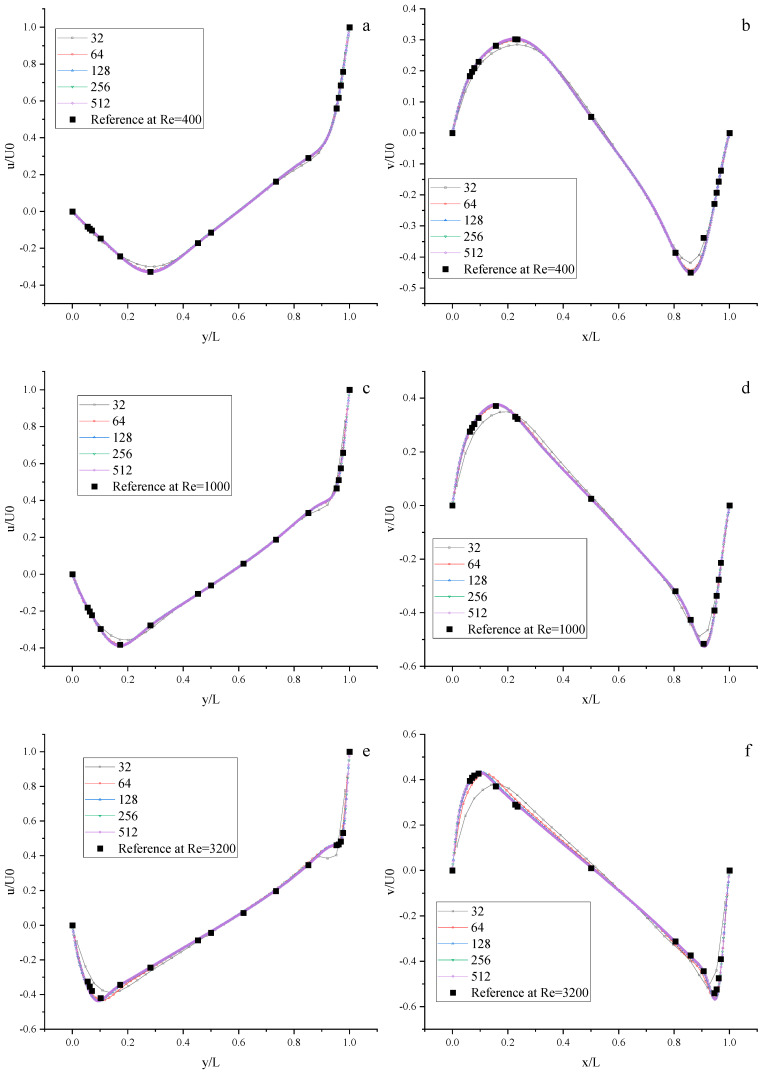
The velocity profiles of the lid-driven cavity flow using the Moment-based scheme. Left: Horizontal velocity along the vertical center line; Right: Vertical velocity along the horizontal center line. (**a**,**b**) Re = 400; (**c**,**d**) Re = 1000; (**e**,**f**) Re = 3200.

**Figure 9:**
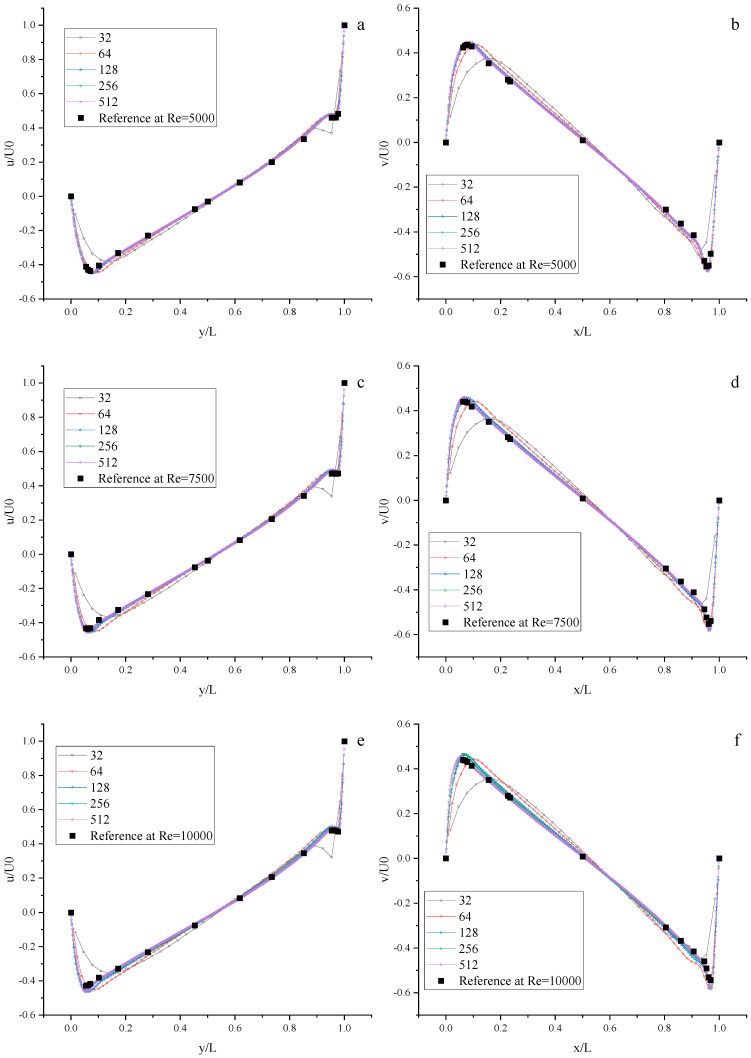
The velocity profiles of the lid-driven cavity flow with the Moment-based scheme. Left: Horizontal velocity along the vertical center line; Right: Vertical velocity along the horizontal center line. (**a**,**b**) Re = 5000; (**c**,**d**) Re = 7500; (**e**,**f**) Re = 10,000.

We perform some numerical simulations using the Moment-based scheme at Re = 400, 1000, 3200, 5000, 7500, and 10,000 with the different uniform meshes (N × N, N = 32, 64, 128, 256, and 512). The results of velocity profiles are present in Figure 8 and Figure 9. It is found that the results of the Moment-based scheme are in good agreement with the reference data [72] with a relatively fine mesh (N = 128, 256, 512).

**Figure 10:**
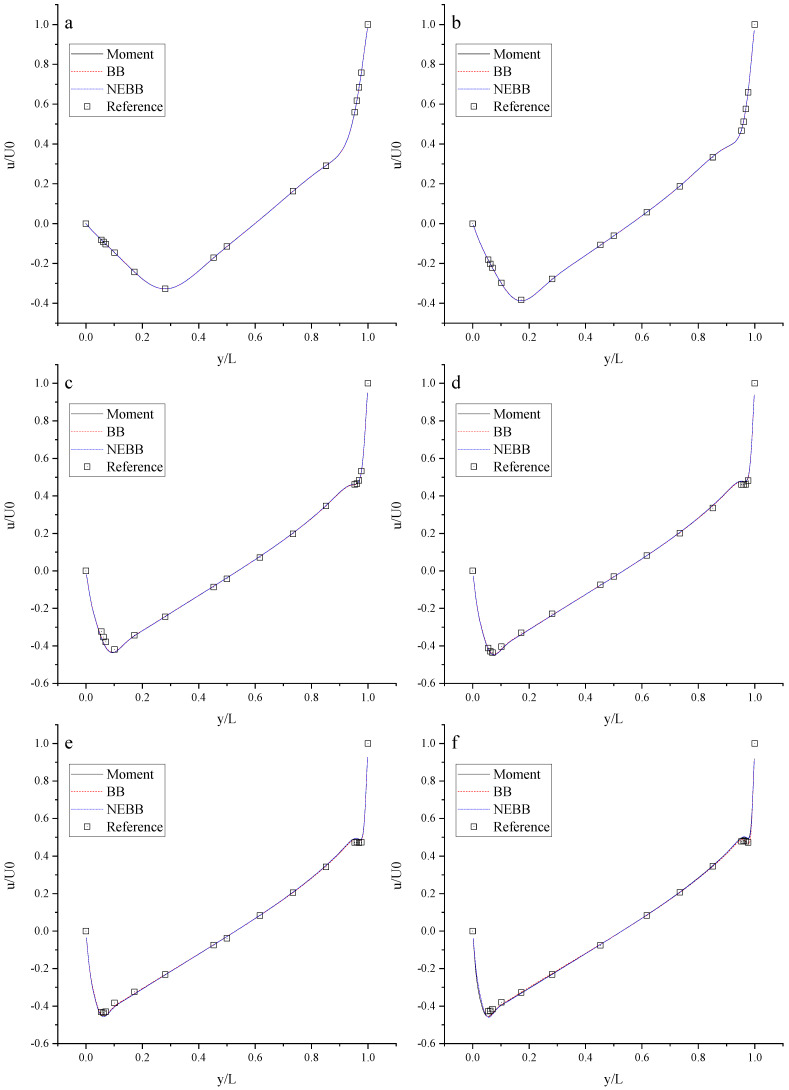
The horizontal velocity along the vertical center line with the present schemes. (**a**) Re = 400; (**b**) Re = 1000; (**c**) Re = 3200; (**d**) Re = 5000; (**e**) Re = 7500; (**f**) Re = 10,000.

**Figure 11:**
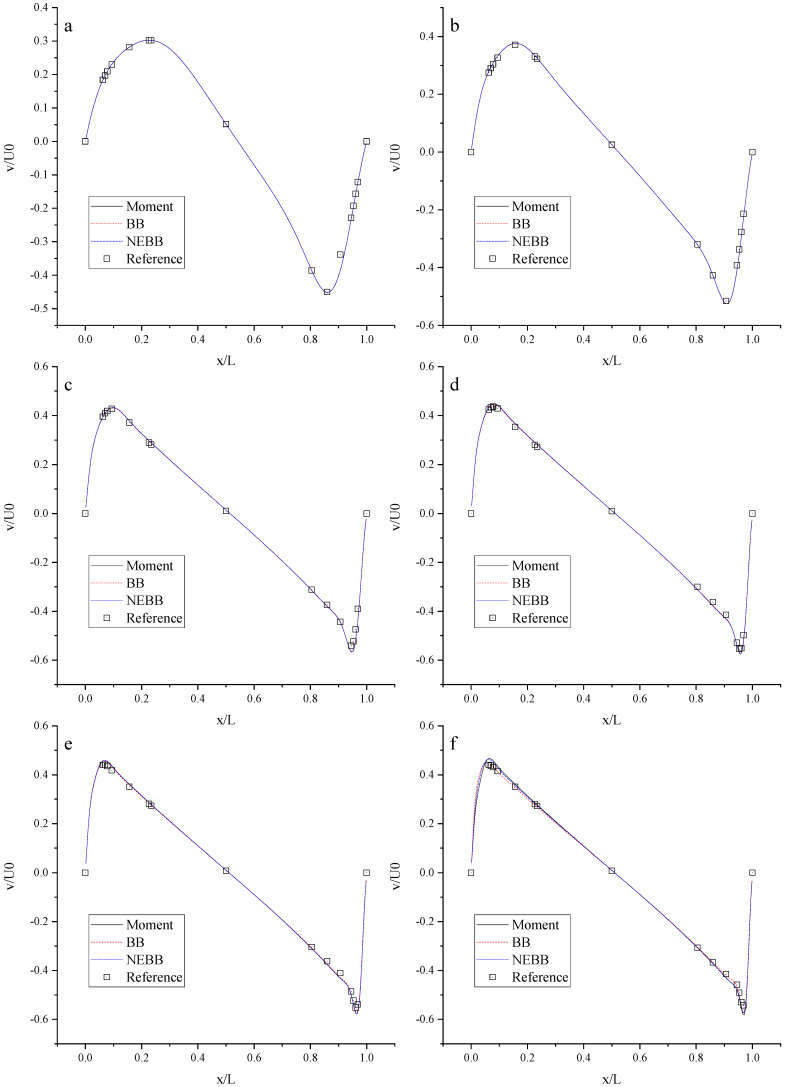
The vertical velocity along the horizontal center line with the present schemes. (**a**) Re = 400; (**b**) Re = 1000; (**c**) Re = 3200; (**d**) Re = 5000; (**e**) Re = 7500; (**f**) Re = 10,000.

To compare the Moment-based scheme with the BB and NEBB scheme, some simulations are performed at Re = 100, 400, 1000, 3200, 5000, 7500, and 10,000 with the fine mesh (N = 256). The results of velocity profiles are present in Figure 10 and Figure 11. It is shown that the results of the Moment-based, BB, and NEBB schemes are all in good agreement with the reference data [72]. Based on a comparison with the reference data, the proposed schemes of the boundary conditions for the DUGKS are valid and suitable for simulating the lid-driven cavity flow.

### 3.3. The Poiseuille Flow

The Poiseuille flow is driven by an external force *ρa_x_* with periodic boundary conditions at the entrance and exit. Both the top and bottom walls adopt the proposed scheme of boundary conditions. The gap between the top and bottom wall is set as *H* = 1. For the no-slip condition, the analytical solution of horizontal velocity in the Poiseuille flow can be expressed as,

*u*(*y*)/*U*max = 4*y*/*H*(1 – *y*/*H*), (63)

where *U*max = *a_x_H*^2^/8*ν*.

It is noted that *a_y_* = 0 and the convergence criterion for simulating the Poiseuille flow follows Equation (60). In this subsection, it is noted that the “Original” scheme represents the boundary condition with *a_x_* = *a_y_* = 0 in Equation (37) or Equation (39), and the “Present” scheme represents the proposed scheme of boundary condition with *a_x_* = *g*, *a_y_* = 0 in Equation (37) or Equation (39).

**Figure 12:**
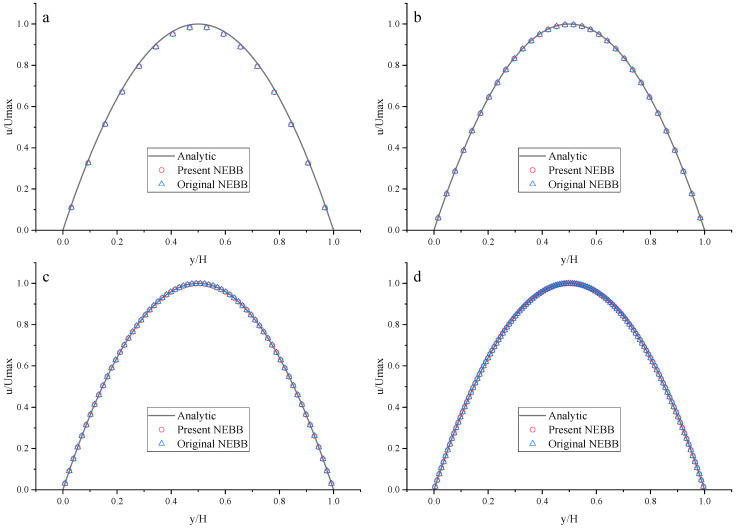
The horizontal velocity profiles with the NEBB schemes in the Poiseuille flow at Re = 100. (**a**) N = 16; (**b**) N = 32; (**c**) N = 64; (**d**) N = 128.

**Figure 13:**
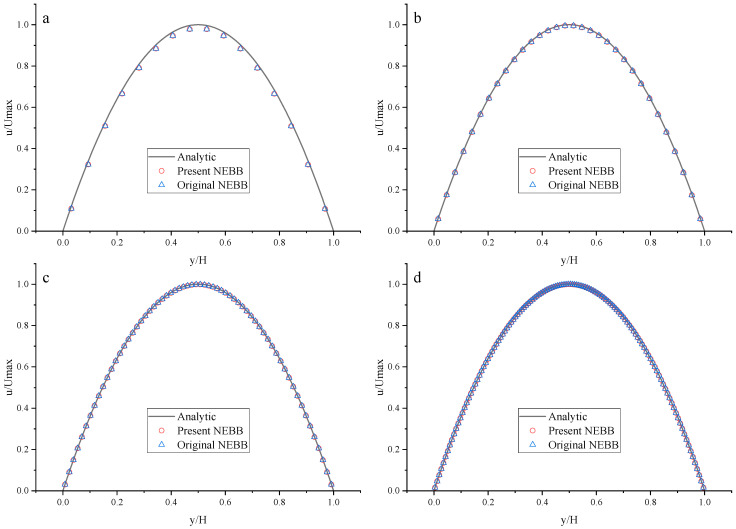
The horizontal velocity profiles with the NEBB schemes in the Poiseuille flow at Re = 1000. (**a**) N = 16; (**b**) N = 32; (**c**) N = 64; (**d**) N = 128.

**Figure 14:**
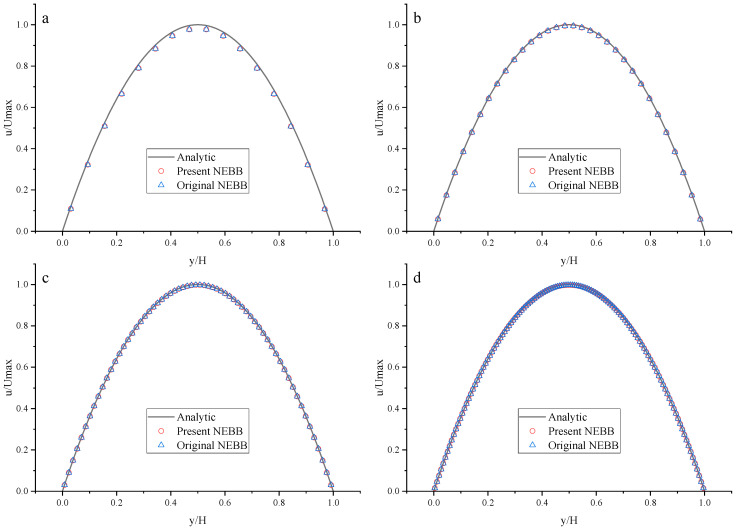
The horizontal velocity profiles with the NEBB schemes in the Poiseuille flow at Re = 10,000. (**a**) N = 16; (**b**) N = 32; (**c**) N = 64; (**d**) N = 128.

To compare the present NEBB scheme (such as Equation (39) with *a_x_* = *g*, *a_y_* = 0) with the original NEBB scheme (such as Equation (39) with *a_x_* = *a_y_* = 0), the original NEBB scheme is also applied to both the top and bottom walls. The results are shown in Figure 12, Figure 13 and Figure 14 and Table 6.

As shown in Figure 12, Figure 13 and Figure 14, it seems that both the present and original NEBB schemes agree very well with the analytical solution with N = 32, 64, and 128 at Re = 100, 1000, and 10,000. Furthermore, the *L*_2_ errors of horizontal velocity are measured. As shown in Table 6, the *L*_2_ error of the present NEBB scheme is less than the original NEBB scheme, which shows the present NEBB scheme is more accurate than the original NEBB scheme. It is found that the present and original NEBB schemes are almost second-order accurate.

**Figure 15:**
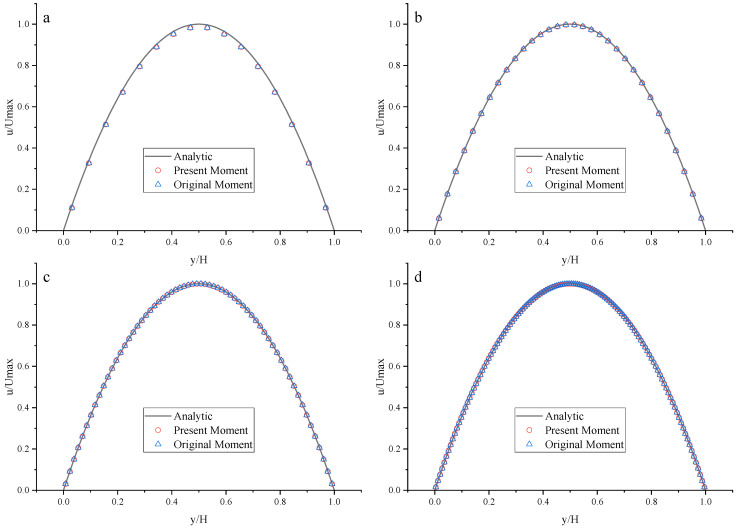
The horizontal velocity profiles with the Moment-based scheme in the Poiseuille flow at Re = 100. (**a**) N = 16; (**b**) N = 32; (**c**) N = 64; (**d**) N = 128.

**Figure 16:**
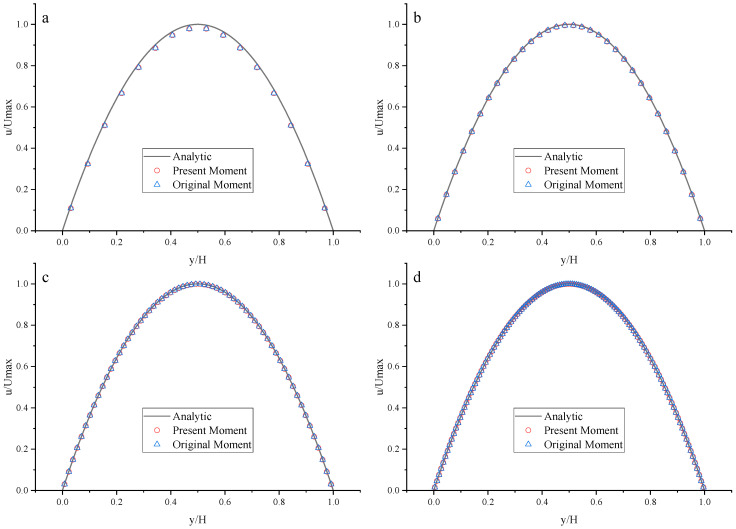
The horizontal velocity profiles with the Moment-based scheme in the Poiseuille flow at Re = 1000. (**a**) N = 16; (**b**) N = 32; (**c**) N = 64; (**d**) N = 128.

**Figure 17:**
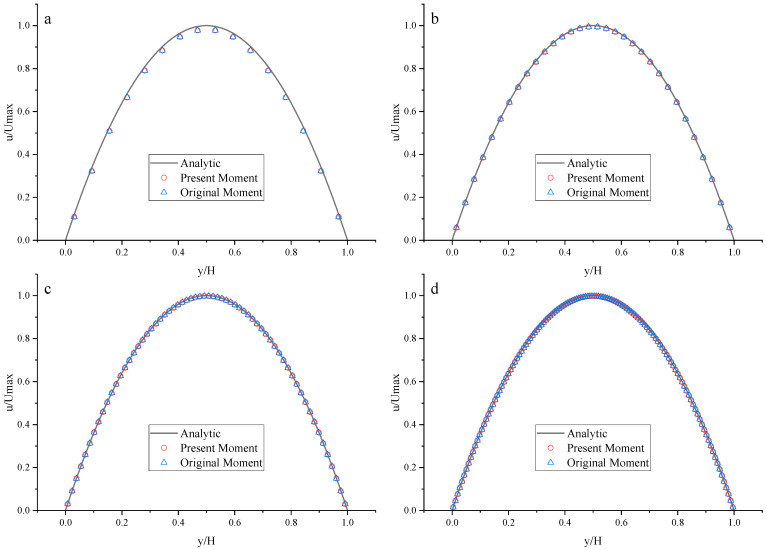
The horizontal velocity profiles with the Moment-based scheme in the Poiseuille flow at Re = 10,000. (**a**) N = 16; (**b**) N = 32; (**c**) N = 64; (**d**) N = 128.

To compare the present Moment-based scheme (such as Equation (37) with *a_x_* = *g*, *a_y_* = 0) with the original Moment-based scheme (such as Equation (37) with *a_x_* = *a_y_* = 0), the original Moment-based scheme is also applied to both top and bottom walls. The results are shown in Figure 15, Figure 16 and Figure 17 and Table 7.

As shown in Figure 15, Figure 16 and Figure 17, it seems that both the present and original Moment-based schemes agree very well with the analytical solution with N = 32, 64, and 128 at Re = 100, 1000, and 10,000. Furthermore, the *L*_2_ errors of horizontal velocity are measured. As shown in Table 7, the *L*_2_ error of the present Moment-based scheme is less than the original Moment-based scheme, which shows the present Moment-based scheme is more accurate than the original Moment-based scheme. It is found that the present and original Moment-based schemes are almost second-order accurate.

Comparing Table 6 with Table 7, it is found that the original Moment-based scheme is more accurate than the original NEBB scheme under different meshes, and the present Moment-based scheme is more accurate than the present NEBB scheme with N = 16 and 32, in contrast to the cases with N = 64 and 128.

To compare the present BB and original and present NEBB and Moment-based schemes with the BB and NEBB schemes proposed by Yang et al. [39], we show the *L*_2_ errors in Table 8.

As shown in Table 8, the original and present schemes can predict more accurate results with a finer mesh. The *L*_2_ errors of the present BB and original and present NEBB schemes are not very sensitive to the meshes, but the *L*_2_ errors of the original and present Moment-based schemes are sensitive to the meshes. The *L*_2_ errors of the present BB and original and present Moment-based schemes are not very sensitive to the kinematic viscosity, but the *L*_2_ errors of the original and present NEBB schemes are very sensitive to the kinematic viscosity. The *L*_2_ errors of the original NEBB scheme are more than those of the present NEBB scheme, which are more than those of the NEBB scheme in Ref. [39]. With a fixed kinematic viscosity and grid number, the *L*_2_ errors of the present Moment-based scheme are minimal.

### 3.4. Normal Dipole–Wall Collision

Two counter-rotating vortices are propelled towards a solid boundary, and they collide with the no-slip boundary. The vortex dipole–wall collision is found in nature, such as the effect of the ground on the formulation of secondary vortices when an airplane takes off or lands [73]; another natural phenomenon is the formulation of large-scale vortices in geophysical turbulence on the coasts of seas and oceans [74]. Therefore, the vortex dipole–wall collision is an important problem.

In this study, two counter-rotating vortices are confined to a square box with a size of [−1, 1] × [−1, 1]. The present boundary conditions are applied to the no-slip walls. The initial vortex is located at positions (*x*_1_, *y*_1_) and (*x*_2_, *y*_2_). The initial velocities are

(64) $$\begin{array}{l} u_{x0}=-0.5\left|We\right|\left(y-y_{1}\right)\exp\left(-{\left(r_{1}/r_{0}\right)}^{2}\right)+0.5\left|We\right|\left(y-y_{2}\right)\exp\left(-{\left(r_{2}/r_{0}\right)}^{2}\right),\\ u_{y0}=0.5\left|We\right|\left(x-x_{1}\right)\exp\left(-{\left(r_{1}/r_{0}\right)}^{2}\right)-0.5\left|We\right|\left(x-x_{2}\right)\exp\left(-{\left(r_{2}/r_{0}\right)}^{2}\right),\\ r_{0}=0.1,\;\;r_{i}=\sqrt{{\left(x-x_{i}\right)}^{2}+{\left(y-y_{i}\right)}^{2}},\;i=1,\;2. \end{array}$$

The symbol *r*_0_ denotes the radius of the monopoles, and *We* represents the strength of the vortices. To compare the numerical results, the total kinetic energy *E*(*t*) and the total enstrophy Ω(*t*) are calculated

(65) $$\begin{array}{l} E(t)=\frac{1}{2}\int_{-1}^{1}\int_{1}^{-1}\left|u^{2}\right|(x,t)dxdy,\\ \Omega (t)=\frac{1}{2}\int_{-1}^{1}\int_{1}^{-1}\left|\omega^{2}\right|(x,t)dxdy,\;\omega =\partial_{x}u_{y}-\partial_{y}u_{x}, \end{array}$$

where *ω* is the vorticity.

Clercx and Bruneau [75] simulated a dipole–wall collision using a Finite Difference Method (FDM) and the Chebyshev Pseudospectral Method (CPM). Mohammed et al. [40] simulated the dipole–wall collision using the LBM with two relaxation time models (TRT-LBM). Their authoritative data will be used as benchmark numerical results. For a direct comparison between the present work and the work of Ref. [40] and Ref. [75], we use the grid number (N) in the DUGKS as follows: Re = 625 (N = 1024), Re = 1250 (N = 1536), Re = 2500 (N = 2048) and Re = 5000 (N = 3072).

**Figure 18:**
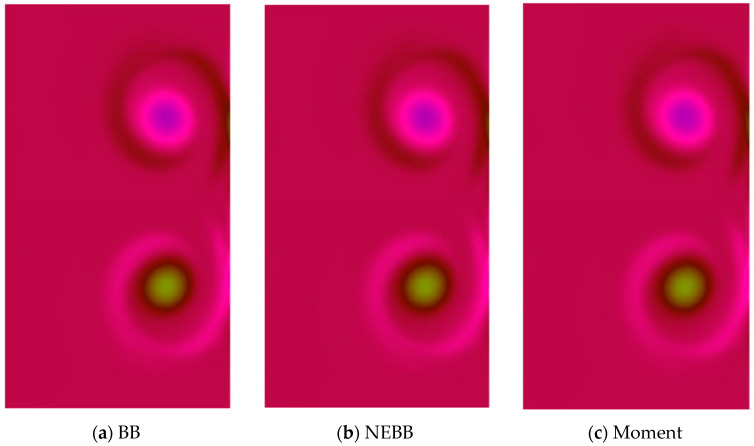
Vorticity contours of normal dipole–wall collision at *t* = 1 and Re = 625 (subdomain: 0.3 ≤ *x* ≤ 1, −0.6 ≤ *x* ≤ 0.6).

**Figure 19:**
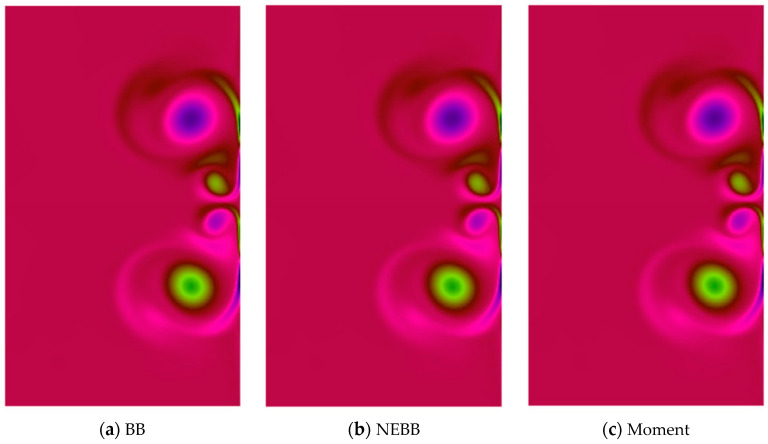
Vorticity contours of normal dipole–wall collision at *t* = 1 and Re = 1250 (subdomain: 0.3 ≤ *x* ≤ 1, −0.6 ≤ *x* ≤ 0.6).

**Figure 20:**
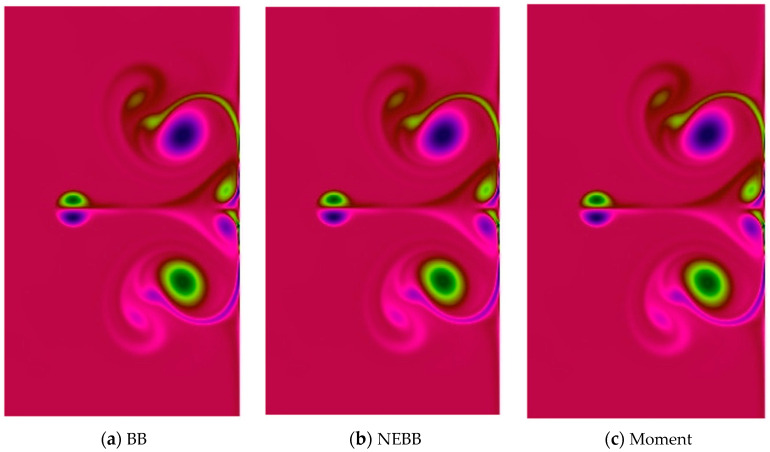
Vorticity contours of normal dipole–wall collision at *t* = 1 and Re = 2500 (subdomain: 0.3 ≤ *x* ≤ 1, −0.6 ≤ *x* ≤ 0.6).

**Figure 21:**
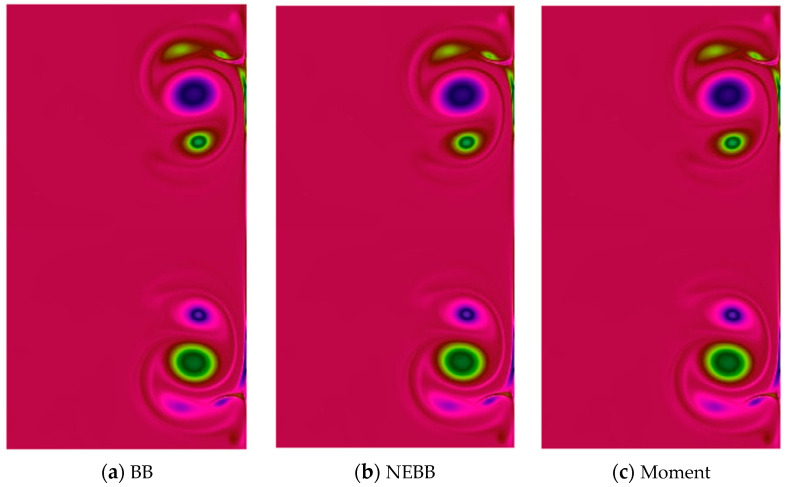
Vorticity contours of normal dipole wall collision at *t* = 1 and Re = 5000 (subdomain: 0.3 ≤ *x* ≤ 1, −0.6 ≤ *x* ≤ 0.6).

In the dipole–wall collision benchmark test for the normal case, two monopoles are located at positions (*x*_1_, *y*_1_) = (0, 0.1) and (*x*_2_, *y*_2_) = (0, −0.1) initially. Then they are propelled towards the right wall. To test the effect of the present schemes on the vortices after the dipole collides, vorticity contour plots are present in Figure 18, Figure 19, Figure 20 and Figure 21. As shown in vorticity contour plots, the present schemes are effective to simulate the dipole–wall collision, but the results with BB, NEBB, and Moment schemes are almost indistinguishable. To analyze the present schemes quantitatively, the values of the first and second maxima enstrophy of dipoles will be grouped for comparisons.

The present results for the first and second local maxima of the enstrophy are shown in Table 9 and Table 10, and we compare them with the results in Refs. [40,75]. The results computed using the moment-based boundary condition with DUGKS and TRT-LBM are in good agreement. However, the data obtained by the DUGKS appear to be more accurate than the data obtained by the TRT-LBM in the sense that the data are in closer agreement with the data obtained by FDM and CPM. The data obtained using the present moment-based scheme appear to be more accurate than the data obtained using the present BB and NEBB schemes in the sense that they are in closer agreement with the data obtained by FDM and CPM. These show that the proposed moment-based scheme can be a competitive method and gives us the confidence to use it to impose physically more complex conditions.

For further comparison with reference data [76], we present the values of the energy *E*(*t*) and enstrophy Ω(*t*) at different times, as shown in Table 11 and Table 12. The results obtained by the D3Q19-CM-LBM [76] show a slight mismatch (up to 3%) with respect to the LBM study by Mohammed et al. [40]. It should be noted that the results obtained by the DUGKS with the present Moment scheme are closer to the reference ones by Clercx and Bruneau [75] than those obtained by the TRT-LBM with the Moment scheme [40]. We address this behavior regarding the adoption in the present work of a more accurate boundary condition.

### 3.5. Rayleigh–Taylor Instability

Rayleigh–Taylor instability can occur when a layer of heavy fluid descends as light fluid below it rises. We perform a simulation using the same parameters as the first case of Re = 256, as shown in Figure 7.4 in Ref. [77]. The left and right boundaries adopt the periodic boundary conditions. For the upper and lower boundaries, the mentioned boundary conditions are applied. A computational domain X × Y = 128 × 512 is employed.

Initially, there is a zero-velocity field, and the location of the perturbed interface is set as *y* = 0.5Y + 0.1Xcos(2π*x*/X), where *x*, *y*, X, and Y are all in lattice units. We set the densities of heavy fluid and light fluid to be *ρ*_h_ = 0.12 and *ρ*_l_ = 0.04, respectively, so that *At* = (*ρ*_h_ − *ρ*_l_)/(*ρ*_h_ + *ρ*_l_) = 0.5. We set *U* = 0.04, and the gravitational acceleration is *g* = *U*^2^/X = 1.25 × 10^−5^ in the -*y* direction.

In this study, the HCZ model [52] is introduced for the DUGKS to simulate the Rayleigh–Taylor instability. In the model, two distribution functions satisfy the following equations:

(66) $$\frac{\partial g}{\partial t}+\xi_{\alpha }\cdot \nabla g=-\frac{1}{\tau_{g}}(g-g^{eq})+S^{g}$$

(67) $$\frac{\partial f}{\partial t}+\xi_{\alpha }\cdot \nabla f=-\frac{1}{\tau_{f}}(f-f^{eq})+S^{f}$$

The distribution functions *g* and *f*, corresponding to the density *ρ* and phase order *ϕ*, denote the particle distribution function in terms of position **x**, discrete particle velocity ***ξ****_α_*, and time *t*. The relaxation times *τ_g_* and *τ_f_* are related to the viscosity and the mobility coefficient in the Cahn–Hilliard equation. Usually, we set *τ_g_* = *τ_f_*.

*S^g^* and *S^f^* represent the source terms, which can be written as

(68) $$S^{g}=(\xi -u)\cdot \left\{\omega (\xi ,u)(F_{s}+\rho a)-[\omega (\xi ,u)-\omega (\xi ,0)]\nabla \psi (\rho )\right\}$$

(69) $$S^{f}=-\omega (\xi ,u)\frac{\left(\xi -u)\cdot \nabla \psi (\phi \right)}{RT}$$

(70) $$\omega (\xi ,u)=\frac{1}{{\left(2\pi RT\right)}^{D/2}}\left[\frac{{\left(\xi -u\right)}^{2}}{2RT}\right]$$

***F****_s_* represents the force associated with surface tension,

(71) $$F_{s}=\kappa \rho \nabla \nabla^{2}\rho$$

where the parameter *κ* determines the strength of surface tension and *κ* = 0.01 in our simulations.

*ψ* represents the function of the density *ρ* or phase order *ϕ.* ψ(ρ) and ψ(ϕ) are determined as [78]

(72) $$\psi (\rho )=p-c_{s}^{2}\rho$$

(73) $$\psi (\phi )=p_{th}-c_{s}^{2}\phi$$

(74) $$p_{th}=\phi c_{s}^{2}\frac{1+b\phi /4+{\left(b\phi /4\right)}^{2}-{\left(b\phi /4\right)}^{3}}{{\left(1-b\phi /4\right)}^{3}}-a\phi^{2}$$

In our simulations, *a =* 12*RT*, *b* = 4.

The equilibrium distributions *g^eq^* and *f^eq^* can be calculated as

(75) $$f^{eq}=\phi \omega (\xi ,u)$$

(76) $$g^{eq}=\rho RT\omega (\xi ,u)+\psi (\rho )\omega (\xi ,0)$$

Since Equations (66) and (67) share the same format as Equation (1), we will not repeat the detailed depiction of evolution in the DUGKS. In this subsection, we study the effect of varied boundary conditions on the numerical simulation results of Rayleigh–Taylor instability.

**Figure 22:**
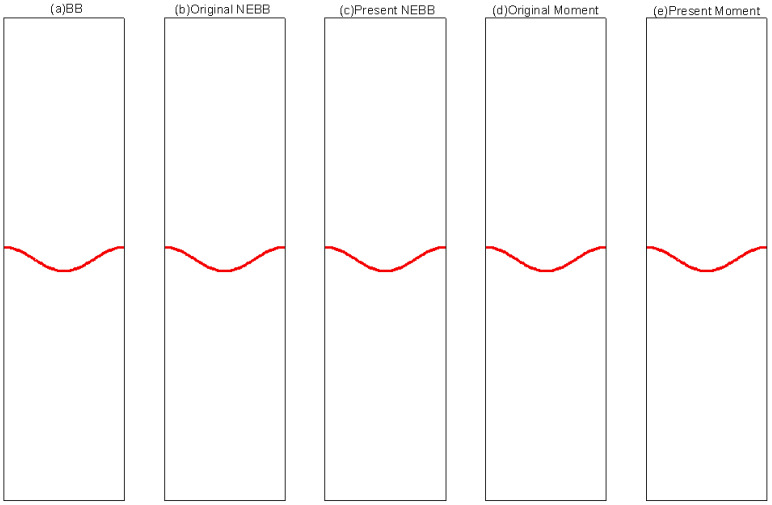
Interface evolution for Rayleigh–Taylor instability simulation with Re = 256 at t* = 0. The non-dimensional time is normalized by (X/*g*)^1/2^.

**Figure 23:**
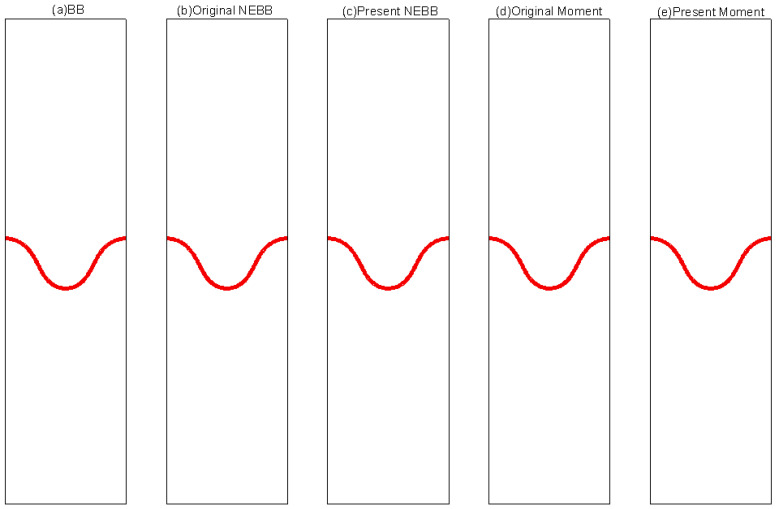
Interface evolution for Rayleigh–Taylor instability simulation with Re = 256 at t* = 1. The non-dimensional time is normalized by (X/*g*)^1/2^.

**Figure 24:**
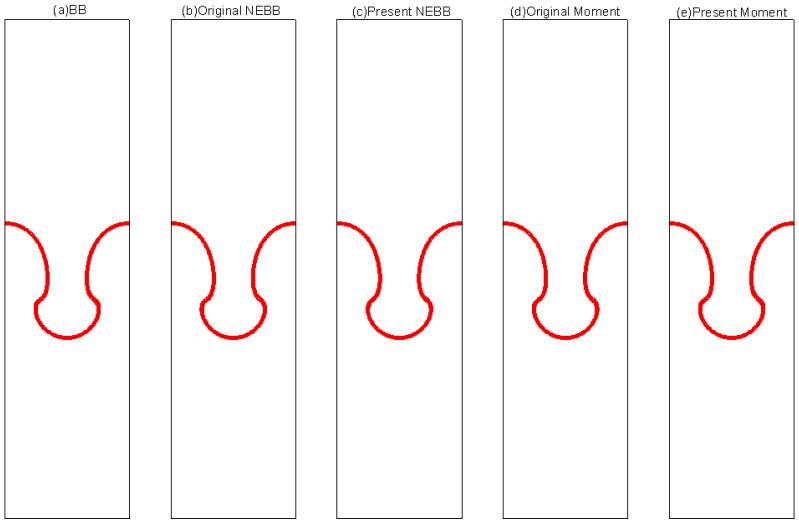
Interface evolution for Rayleigh–Taylor instability simulation with Re = 256 at t* = 2. The non-dimensional time is normalized by (X/*g*)^1/2^.

**Figure 25:**
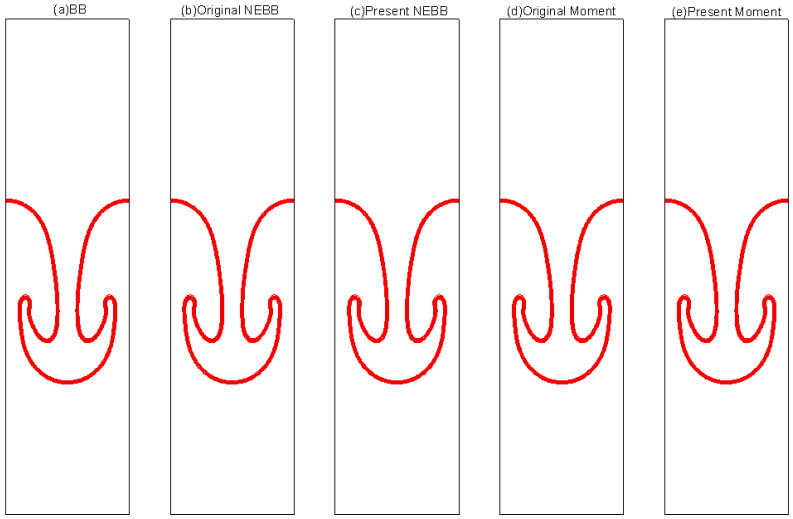
Interface evolution for Rayleigh–Taylor instability simulation with Re = 256 at t* = 3. The non-dimensional time is normalized by (X/*g*)^1/2.^

**Figure 26:**
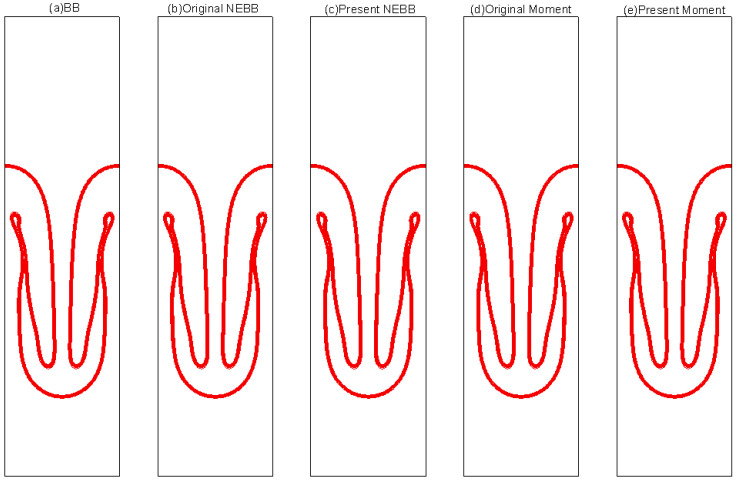
Interface evolution for Rayleigh–Taylor instability simulation with Re = 256 at t* = 4. The non-dimensional time is normalized by (X/*g*)^1/2^.

**Figure 27:**
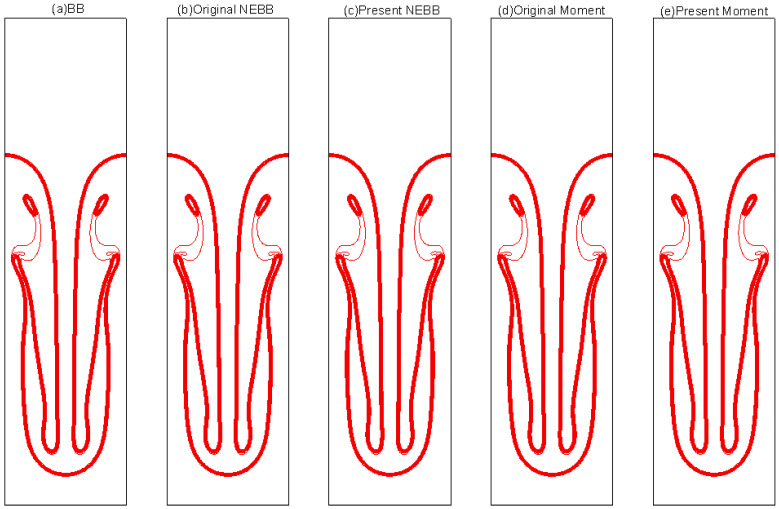
Interface evolution for Rayleigh–Taylor instability simulation with Re = 256 at t* = 5. The non-dimensional time is normalized by (X/*g*)^1/2^.

The results of varied boundary conditions at Re = 256 are shown in Figure 22, Figure 23, Figure 24, Figure 25, Figure 26 and Figure 27. Fifteen equal-interval contours *ρ* = 0.045, 0.05, 0.055, …, 0.115 are drawn in each figure. In this subsection, it is noted that the “Original” scheme represents the boundary condition with *a_x_* = *a_y_* = 0 in Equation (37) or Equation (39), and the “Present” scheme represents the proposed scheme of the boundary condition with *a_x_* = 0, *a_y_* = -*g* in Equation (37) or Equation (39).

As shown in Figure 22, Figure 23, Figure 24, Figure 25, Figure 26 and Figure 27, the fluid interface does not diffuse in our simulations by applying the mentioned boundary conditions. Compared to Figure 7.4 in Ref. [77], the evolution of the interface is indistinguishable from the results computed on the same mesh. It is shown that the present schemes are accurate and stable to simulate the Rayleigh–Taylor instability.

**Figure 28:**
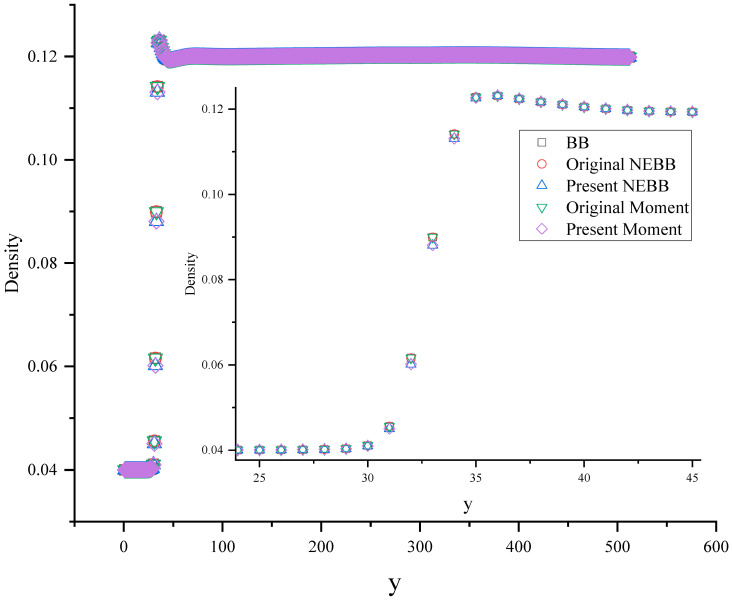
Density profiles across the spike at t* = 5. The horizontal axis is the computational grid.

**Figure 29:**
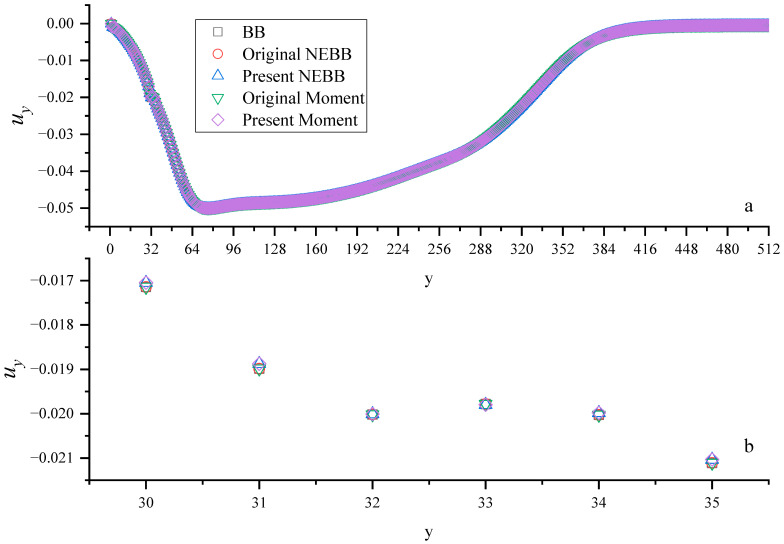
Vertical velocity profiles across the spike at t* = 5, (**b**) is the magnified view of (**a**). The horizontal axis is the computational grid.

Figure 28 and Figure 29 show the density and vertical velocity profiles across the spike, respectively. As shown in Figure 28 and Figure 29, the interface thickness takes approximately four grid spacings (y = 31, 32, 33, and 34). There exist some “jiggles” near the interface, and the “jiggles” are different due to using different boundary conditions. From the magnified view in Figure 28 and Figure 29, the density and absolute value of vertical velocity near the interface using present NEBB and Moment-based schemes are less than that using BB, original NEBB, and original Moment-based schemes. Although the interface thickness is not affected by different boundary conditions, choosing a wall boundary condition can influence the density and velocity near the interface. Usually, numerical instability occurs near the interface. So, the finding reminds us that wall boundary conditions should be treated with care.

The simulation is executed on a Win10 X64 system with Intel(R) Core(TM) i5-8250U CPU (1.60 GHz)s. For comparison, the convergence error and CPU time at the last time step are recorded in Table 13. As shown in Table 13, the *error* and CPU time of the present Moment-based scheme are minimal, although they are almost indistinguishable.

## 4. Conclusions

Owing to the DUGKS with transformed distribution functions and force terms, we should convert the original boundary conditions into a new format. In this study, the proposed schemes of the BB, NEBB, and Moment-based boundary conditions are proposed for the DUGKS. The boundary conditions will be translated into constraints on the unknown transformed distribution functions at a half time step (*t* + 0.5Δ*t*). The present work tests and analyzes, for the first time, the Moment-based scheme for the DUGKS. The mentioned boundary conditions are evaluated through theoretical analysis and numerical tests.

Using the steady unidirectional flow, we theoretically analyze the numerical slip error of the present BB, NEBB, and Moment-based schemes. It is found that the numerical slip errors of both NEBB and Moment-based schemes are equal to zero, which implements the no-slip condition at the wall boundary.

The accuracy and stability of the present schemes are validated by numerical tests of Couette flow, Poiseuille flow, Lid-driven cavity flow, normal dipole–wall collision, and Rayleigh–Taylor instability. The following conclusions are obtained:

(1) Couette flow

The present schemes can predict accurate results of simulating the Couette flow, even with a coarse mesh for a large Reynolds number (Re = 10^6^). The *L*_2_ errors in both NEBB and Moment-based schemes are equal, which are a little less than those in the BB scheme at Re = 100 under different meshes. Both the NEBB and Moment-based schemes are more accurate and converge to the steady state faster than the BB scheme at high Re (Re = 100,000, 1,000,000, and 10,000,000).

(2) Lid-driven cavity flow

The present schemes can predict accurate results by simulating the lid-driven cavity flow. It is found that the results of the BB scheme are in better agreement with the reference data than those of Moment and NEBB schemes at Re = 10,000.

(3) Poiseuille flow

The results of the present schemes agree very well with the analytical solution under different meshes (N = 32, 64, 128) at Re = 100, 1000, and 10,000. The *L*_2_ errors of the present NEBB and Moment-based schemes are less than the original NEBB and Moment-based schemes, respectively, which shows the present schemes are more accurate than the original schemes. It is found that the present schemes for the DUGKS are second-order accurate. The original Moment scheme is more accurate than the original NEBB scheme under different meshes. The present Moment scheme is more accurate than the present NEBB scheme with N = 16 and 32, in contrast to the cases with N = 64 and 128. Compared with the BB and NEBB schemes in Ref. [39], the *L*_2_ errors of the present Moment-based scheme are minimal.

(4) Normal dipole–wall collision

With the present moment-based scheme, the DUGKS appears to be more accurate than the TRT-LBM. The data obtained by using the present moment-based scheme appears to be more accurate than the data obtained by using the present BB and NEBB schemes in the sense that they are in closer agreement with the benchmark data. These show that the proposed moment-based scheme can be a competitive method.

(5) Rayleigh–Taylor instability

The present results agree well with the reference results, which show the present schemes can effectively capture the evolution of the interface. It is found that the error and CPU time of the present Moment-based scheme are minimal among the mentioned boundary conditions. The Rayleigh–Taylor instability simulation shows that choosing a wall boundary condition can influence the density and velocity near the interface slightly.

## Figures and Tables

**Table 1:** The *L*_2_ errors of the horizontal velocity of the Couette flow at Re = 100 (*τ* = 0.003).

**N**	128	96	64	32	16	8
**BB**	1.66628 × 10^−13^	1.27405 × 10^−6^	3.81605 × 10^−6^	8.79265 × 10^−6^	1.32854 × 10^−5^	1.72066 × 10^−5^
**NEBB**	1.27816 × 10^−13^	1.27338 × 10^−6^	3.81166 × 10^−6^	8.78087 × 10^−6^	1.32812 × 10^−5^	1.71994 × 10^−5^
**Moment**	1.27816 × 10^−13^	1.27338 × 10^−6^	3.81166 × 10^−6^	8.78087 × 10^−6^	1.32812 × 10^−5^	1.71994 × 10^−5^

**Table 2:** The *L*_2_ errors of the horizontal velocity of the Couette flow at Re = 1000 (*τ* = 0.0003).

**N**	128	96	64	32	16	8
**BB**	9.71675 × 10^−11^	2.1845 × 10^−5^	4.52773 × 10^−5^	9.18423 × 10^−5^	1.37823 × 10^−4^	1.84314 × 10^−4^
**NEBB**	1.09616 × 10^−10^	2.2389 × 10^−5^	4.51525 × 10^−5^	9.17737 × 10^−5^	1.38751 × 10^−4^	1.84293 × 10^−4^
**Moment**	1.09616 × 10^−10^	2.2389 × 10^−5^	4.51525 × 10^−5^	9.17737 × 10^−5^	1.38751 × 10^−4^	1.84293 × 10^−4^

**Table 3:** The *L*_2_ errors of the horizontal velocity of the Couette flow at Re = 10,000 (*τ* = 0.00003).

**N**	128	96	64	32	16	8
**BB**	1.22165 × 10^−8^	0.000228773	0.000462571	0.000926131	0.001389311	0.001852818
**NEBB**	1.21692 × 10^−8^	0.000229457	0.000462466	0.000926677	0.001390013	0.001852627
**Moment**	1.21692 × 10^−8^	0.000229457	0.000462466	0.000926677	0.001390013	0.001852627

**Table 4:** The steps of reaching the steady state.

**Re**	10^5^	10^6^	10^7^
**BB**	13,936,000	84,718,000	341,489,000
**NEBB**	13,912,000	84,530,000	340,096,000
**Moment**	13,912,000	84,530,000	340,096,000

**Table 5:** The *L*_2_ errors of the horizontal velocity of the Couette flow at high Re with N = 16.

**Re**	10^5^	10^6^	10^7^
**BB**	0.002295662	0.022480202	0.185716773
**NEBB**	0.002293253	0.02246215	0.185606295
**Moment**	0.002293253	0.02246215	0.185606295

**Table 6:** *L*2 errors of horizontal velocity profiles with the present and original NEBB schemes.

	Re	N	16	32	64	128
Present NEBB	100	E(u)	0.018755994	0.003825606	0.000710039	0.000136376
		Order	-	2.293591505	2.429718642	2.380306222
	1000	E(u)	0.023801505	0.00582911	0.001413665	0.000454996
		Order	-	2.02970529	2.043835553	1.635515118
	10,000	E(u)	0.024690217	0.00658069	0.002407783	0.002264078
		Order	-	1.907628779	1.450533728	0.088781481
Original NEBB	100	E(u)	0.019112955	0.004009596	0.000801462	0.000181186
		Order	-	2.253021976	2.3227516	2.145163806
	1000	E(u)	0.024169781	0.006020357	0.00150967	0.000503777
		Order	-	2.005283422	1.995616279	1.583376133
	10,000	E(u)	0.025058973	0.006773108	0.002507213	0.002314213
		Order	-	1.887437355	1.433733542	0.115563095

**Table 7:** *L*2 errors of horizontal velocity profiles with the Moment-based schemes.

	Re	N	16	32	64	128
Present Moment	100	E(u)	0.017994738	0.003719606	0.000723119	0.00015594
		Order	-	2.274353273	2.362844288	2.213239174
	1000	E(u)	0.023096898	0.005750074	0.00144144	0.000486723
		Order	-	2.006046599	1.996069655	1.566337141
	10,000	E(u)	0.023992289	0.006505959	0.002441431	0.002298699
		Order	-	1.882737218	1.414034827	0.086909255
Original Moment	100	E(u)	0.017880081	0.003662118	0.000694374	0.000141598
		Order	-	2.287603033	2.398892803	2.293909228
	1000	E(u)	0.023085474	0.005744352	0.001438569	0.000485323
		Order	-	2.006769196	1.997509524	1.567617086
	10,000	E(u)	0.023991094	0.006505425	0.002441139	0.002298563
		Order	-	1.882783739	1.414088594	0.086822289

**Table 8:** *L*_2_ errors of the present BB, original and present NEBB and Moment-based schemes, and reference schemes proposed by Yang et al. [39].

Kinematic Viscosity *ν*	Gird Number N	Present BB	Original NEBB	Present NEBB	Original Moment	Present Moment	BB [39]	NEBB [39]
0.1	10	0.718039588	0.138149973	0.136013504	0.031323870	0.018755201	0.7231	0.0268
0.1	20	0.686509065	0.127035390	0.125775101	0.004820268	0.004451455	0.7182	0.0068
0.01	20	0.226712876	0.006759553	0.006523803	0.005189780	0.004274516	0.0106	0.0050

**Table 9:** The values of the first maxima enstrophy Ω(*t*) of the dipole.

Re	DUGKS with Present Schemes			Mohammed et al. (TRT-LBM) [40]		Clercx and Bruneau [75]	
	BB	NEBB	Moment	BB	Moment	FDM	CPM
625	889.6	926.2	933.1	853.7	931.6	932.8	933.6
1250	1835	1861	1892	1752	1884	1891	1899
2500	3098	3157	3306	2993	3305	3270	3313
5000	5234	5346	5499	4975	5496	5435	5536

**Table 10:** The values of the second maxima enstrophy Ω(*t*) of the dipole.

Re	DUGKS with Present Schemes			Mohammed et al. (TRT-LBM) [40]		Clercx and Bruneau [75]	
	BB	NEBB	Moment	BB	Moment	FDM	CPM
625	300.4	302.6	305.5	297.5	306.2	305.2	305.2
1250	708.7	714.8	725.8	705.4	727.5	724.9	725.3
2500	1377	1397	1412	1352	1413	1408	1418
5000	3423	3586	3701	3394	3702	3667	3733

**Table 11:** Energy *E*(*t*) at salient time instants (*t* = 0.25, 0.50, 0.75).

Re	*t*	TRT-LBM with Moment Scheme [40]	TRT-LBM with Moment Scheme [40]	FDM [75]	D3Q19- CM-LBM [76]
625	0.25	1.502	1.501	1.502	1.494
	0.50	1.013	1.013	1.013	1.010
	0.75	0.767	0.767	0.767	0.765
1250	0.25	1.720	1.719	1.721	1.710
	0.50	1.312	1.312	1.313	1.308
	0.75	1.061	1.061	1.061	1.057
2500	0.25	1.850	1.848	1.851	1.838
	0.50	1.541	1.540	1.541	1.534
	0.75	1.326	1.325	1.326	1.320

**Table 12:** Enstrophy Ω(*t*) at salient time instants (*t* = 0.25, 0.50, 0.75).

Re	*t*	DUGKS with Present Moment Scheme	TRT-LBM with Moment Scheme [40]	FDM [75]	D3Q19- CM-LBM [76]
625	0.25	472.5	472.1	472.7	467.2
	0.50	381.1	382.6	380.6	374.0
	0.75	255.6	256.0	255.0	244.8
1250	0.25	615.5	613.6	615.0	603.6
	0.50	611.7	612.8	611.3	601.7
	0.75	484.0	486.2	484.4	473.1
2500	0.25	727.1	725.6	727.8	705.3
	0.50	917.2	917.6	916.6	898.1
	0.75	806.1	809.9	805.5	790.2

**Table 13:** The convergence error and CPU time at the last time step *n* = 16,000 (Re = 256).

Boundary Condition	Error	CPU Time
BB	2.784937 × 10^−4^	2671.988
Original NEBB	2.784937 × 10^−4^	2702.598
Present NEBB	2.784777 × 10^−4^	2701.447
Original Moment	2.784937 × 10^−4^	2700.497
Present Moment	2.784776 × 10^−4^	2669.315

## Data Availability

The data that support the findings of this study are available within the article.

## Appendix A. Derivations for the Novel Schemes

Here the derivation process of the moment constraints that the transformed distribution functions ${\bar{f}}_{i}$ obeys *i* the present schemes is shown.

For the south boundary (*u_wy_* = 0), the unknown distribution functions are ${\bar{f}}_{2}$, ${\bar{f}}_{5}$, and ${\bar{f}}_{6}$.

In the moment-based scheme, the moment constraints are:

(A1) $$\Pi_{x}=\rho u_{wx},\;\Pi_{y}=0,\;\Pi_{xx}=\Pi_{xx}^{\left(0\right)}=\rho /3+\rho u_{wx}^{2}$$

With the force term, expanding the density and the moment constraints (A1) yields the system:

(A2) $$\begin{array}{l} \rho ={\bar{f}}_{0}+{\bar{f}}_{1}+{\bar{f}}_{2}+{\bar{f}}_{3}+{\bar{f}}_{4}+{\bar{f}}_{5}+{\bar{f}}_{6}+{\bar{f}}_{7}+{\bar{f}}_{8}\\ \prod_{x}={\bar{f}}_{1}-{\bar{f}}_{3}+{\bar{f}}_{5}-{\bar{f}}_{6}-{\bar{f}}_{7}+{\bar{f}}_{8}+0.5\rho a_{x}h=\rho u_{wx}\\ \prod_{y}={\bar{f}}_{2}-{\bar{f}}_{4}+{\bar{f}}_{5}+{\bar{f}}_{6}-{\bar{f}}_{7}-{\bar{f}}_{8}+0.5\rho a_{y}h=0\\ \prod_{xx}={\bar{f}}_{1}+{\bar{f}}_{3}+{\bar{f}}_{5}+{\bar{f}}_{6}+{\bar{f}}_{7}+{\bar{f}}_{8}=\rho /3+\rho u_{wx}^{2} \end{array}$$

Putting all the unknowns on the left-hand side, the system of equations takes the following form:

(A3) $$\begin{array}{l} {\bar{f}}_{2}+{\bar{f}}_{5}+{\bar{f}}_{6}=\rho -{\bar{f}}_{0}-{\bar{f}}_{1}-{\bar{f}}_{3}-{\bar{f}}_{4}-{\bar{f}}_{7}-{\bar{f}}_{8}\\ {\bar{f}}_{5}-{\bar{f}}_{6}=\rho u_{wx}-{\bar{f}}_{1}+{\bar{f}}_{3}+{\bar{f}}_{7}-{\bar{f}}_{8}-0.5\rho a_{x}h\\ {\bar{f}}_{2}+{\bar{f}}_{5}+{\bar{f}}_{6}={\bar{f}}_{4}+{\bar{f}}_{7}+{\bar{f}}_{8}-0.5\rho a_{y}h\\ {\bar{f}}_{5}+{\bar{f}}_{6}=\rho /3+\rho u_{wx}^{2}-{\bar{f}}_{1}-{\bar{f}}_{3}-{\bar{f}}_{7}-{\bar{f}}_{8} \end{array}$$

Solving the system (A3) yields the unknown distribution functions in the moment-based scheme:

(A4) $$\begin{array}{l} {\bar{f}}_{2}={\bar{f}}_{1}+{\bar{f}}_{3}+{\bar{f}}_{4}+2({\bar{f}}_{7}+{\bar{f}}_{8})-\rho /3-\rho u_{wx}^{2}-0.5\rho a_{y}h\\ {\bar{f}}_{5}=\rho /6-{\bar{f}}_{1}-{\bar{f}}_{8}+0.5\rho u_{wx}^{2}+0.5\rho u_{wx}-0.25\rho a_{x}h\\ {\bar{f}}_{6}=\rho /6-{\bar{f}}_{3}-{\bar{f}}_{7}+0.5\rho u_{wx}^{2}-0.5\rho u_{wx}+0.25\rho a_{x}h\\ \rho =[{\bar{f}}_{0}+{\bar{f}}_{1}+{\bar{f}}_{3}+2({\bar{f}}_{4}+{\bar{f}}_{7}+{\bar{f}}_{8})]/(1+0.5a_{y}h) \end{array}$$

In the NEBB scheme, the moment constraints are:

(A5) $$\Pi_{x}=\rho u_{wx},\;\Pi_{y}=0,\;Q_{xxy}=0.$$

With the force term, expanding the density and the moment constraints (A5) yields the system:

(A6) $$\begin{array}{l} \rho ={\bar{f}}_{0}+{\bar{f}}_{1}+{\bar{f}}_{2}+{\bar{f}}_{3}+{\bar{f}}_{4}+{\bar{f}}_{5}+{\bar{f}}_{6}+{\bar{f}}_{7}+{\bar{f}}_{8}\\ \prod_{x}={\bar{f}}_{1}-{\bar{f}}_{3}+{\bar{f}}_{5}-{\bar{f}}_{6}-{\bar{f}}_{7}+{\bar{f}}_{8}+0.5\rho a_{x}h=\rho u_{wx}\\ \prod_{y}={\bar{f}}_{2}-{\bar{f}}_{4}+{\bar{f}}_{5}+{\bar{f}}_{6}-{\bar{f}}_{7}-{\bar{f}}_{8}+0.5\rho a_{y}h=0\\ Q_{xxy}={\bar{f}}_{5}+{\bar{f}}_{6}-{\bar{f}}_{7}-{\bar{f}}_{8}=0 \end{array}$$

Putting all the unknowns on the left-hand side, the system of equations takes the following form:

(A7) $$\begin{array}{l} {\bar{f}}_{2}+{\bar{f}}_{5}+{\bar{f}}_{6}=\rho -{\bar{f}}_{0}-{\bar{f}}_{1}-{\bar{f}}_{3}-{\bar{f}}_{4}-{\bar{f}}_{7}-{\bar{f}}_{8}\\ {\bar{f}}_{5}-{\bar{f}}_{6}=\rho u_{wx}-{\bar{f}}_{1}+{\bar{f}}_{3}+{\bar{f}}_{7}-{\bar{f}}_{8}-0.5\rho a_{x}h\\ {\bar{f}}_{2}+{\bar{f}}_{5}+{\bar{f}}_{6}={\bar{f}}_{4}+{\bar{f}}_{7}+{\bar{f}}_{8}-0.5\rho a_{y}h\\ {\bar{f}}_{5}+{\bar{f}}_{6}={\bar{f}}_{7}+{\bar{f}}_{8} \end{array}$$

Solving the system (A7) yields the unknown distribution functions in the NEBB scheme:

(A8) $$\begin{array}{l} {\bar{f}}_{2}={\bar{f}}_{4}-0.5\rho a_{y}h\\ {\bar{f}}_{5}={\bar{f}}_{7}-({\bar{f}}_{1}-{\bar{f}}_{3})/2+0.5\rho u_{wx}-0.25\rho a_{x}h\\ {\bar{f}}_{6}={\bar{f}}_{8}+({\bar{f}}_{1}-{\bar{f}}_{3})/2-0.5\rho u_{wx}+0.25\rho a_{x}h\\ \rho =[{\bar{f}}_{0}+{\bar{f}}_{1}+{\bar{f}}_{3}+2({\bar{f}}_{4}+{\bar{f}}_{7}+{\bar{f}}_{8})]/(1+0.5a_{y}h) \end{array}$$

In the BB scheme, the moment constraints are:

(A9) $$\Pi_{y}=0,\;Q_{xyy}=\rho u_{wx}/3,\;Q_{xxy}=0.$$

With the force term, expanding the density and the moment constraints (A9) yields the system:

(A10) $$\begin{array}{l} \rho ={\bar{f}}_{0}+{\bar{f}}_{1}+{\bar{f}}_{2}+{\bar{f}}_{3}+{\bar{f}}_{4}+{\bar{f}}_{5}+{\bar{f}}_{6}+{\bar{f}}_{7}+{\bar{f}}_{8}\\ \prod_{y}={\bar{f}}_{2}-{\bar{f}}_{4}+{\bar{f}}_{5}+{\bar{f}}_{6}-{\bar{f}}_{7}-{\bar{f}}_{8}+0.5\rho a_{y}h=0\\ Q_{xyy}={\bar{f}}_{5}+{\bar{f}}_{8}-{\bar{f}}_{6}-{\bar{f}}_{7}=\rho u_{wx}/3\\ Q_{xxy}={\bar{f}}_{5}+{\bar{f}}_{6}-{\bar{f}}_{7}-{\bar{f}}_{8}=0 \end{array}$$

Putting all the unknowns on the left-hand side, the system of equations takes the following form:

(A11) $$\begin{array}{l} {\bar{f}}_{2}+{\bar{f}}_{5}+{\bar{f}}_{6}=\rho -{\bar{f}}_{0}-{\bar{f}}_{1}-{\bar{f}}_{3}-{\bar{f}}_{4}-{\bar{f}}_{7}-{\bar{f}}_{8}\\ {\bar{f}}_{2}+{\bar{f}}_{5}+{\bar{f}}_{6}={\bar{f}}_{4}+{\bar{f}}_{7}+{\bar{f}}_{8}-0.5\rho a_{y}h\\ {\bar{f}}_{5}-{\bar{f}}_{6}={\bar{f}}_{7}-{\bar{f}}_{8}+\rho u_{wx}/3\\ {\bar{f}}_{5}+{\bar{f}}_{6}={\bar{f}}_{7}+{\bar{f}}_{8} \end{array}$$

Solving the system (A11) yields the unknown distribution functions in the BB scheme:

(A12) $$\begin{array}{l} {\bar{f}}_{2}={\bar{f}}_{4}-0.5\rho a_{y}h\\ {\bar{f}}_{5}={\bar{f}}_{7}+\rho u_{wx}/6\\ {\bar{f}}_{6}={\bar{f}}_{8}-\rho u_{wx}/6\\ \rho =[{\bar{f}}_{0}+{\bar{f}}_{1}+{\bar{f}}_{3}+2({\bar{f}}_{4}+{\bar{f}}_{7}+{\bar{f}}_{8})]/(1+0.5a_{y}h) \end{array}$$
